# Genomic biomarkers of immunotherapy plus chemotherapy in patients with advanced NSCLC: Insights from the phase 3 ORIENT-11 study

**DOI:** 10.1016/j.isci.2026.114730

**Published:** 2026-01-19

**Authors:** Jun Liao, Jie Huang, Xueyuan Chen, Shaodong Hong, Gang Chen, Yaxiong Zhang, Ting Zhou, Weitao Zhuang, Lanlan Pang, Yunpeng Yang, Li Zhang, Wenfeng Fang

**Affiliations:** 1 Department of Medical Oncology, Sun Yat-sen University Cancer Center, State Key Laboratory of Oncology in South China, Collaborative Innovation Center for Cancer Medicine, Guangdong Provincial Clinical Research Center for Cancer, Guangzhou, China; 2 Department of Pulmonary and Critical Care Medicine, Guangzhou Institute of Respiratory Health, The First Affiliated Hospital of Guangzhou Medical University, Guangzhou, China

**Keywords:** Oncology, Therapeutics

## Abstract

To optimize patient selection for first-line immune-checkpoint inhibitor plus chemotherapy (ICI-Chemo) in advanced non-small cell lung cancer (NSCLC), we developed a 9-gene Immune-Chemotherapy Prediction Score (ICPscore) from the phase 3 ORIENT-11 trial. A high ICPscore identified patients with markedly superior progression-free and overall survival from ICI-Chemo versus chemotherapy alone, whereas low-scorers derived minimal benefit, outperforming existing biomarkers such as PD-L1. Its predictive value for immunotherapy efficacy was further validated in the OAK (NSCLC) and IMvigor210 (metastatic urothelial cancer) cohorts. Multi-cohort and single-cell analyses linked a high ICPscore to an immune-active tumor microenvironment characterized by myeloid cell activation, thus providing a biological rationale for its enhanced performance and positioning it as a robust tool for treatment personalization.

## Highlights

• 9-gene ICPscore predicts survival benefit of ICI plus chemo in advanced NSCLC
• High ICPscore patients benefit from ICI plus chemo; low scorers do not
• ICPscore validated in independent cohorts and outperforms PD-L1 expression
• ICPscore reflects an immune-active tumor microenvironment with myeloid activation

## Graphical abstract

**Figure:**
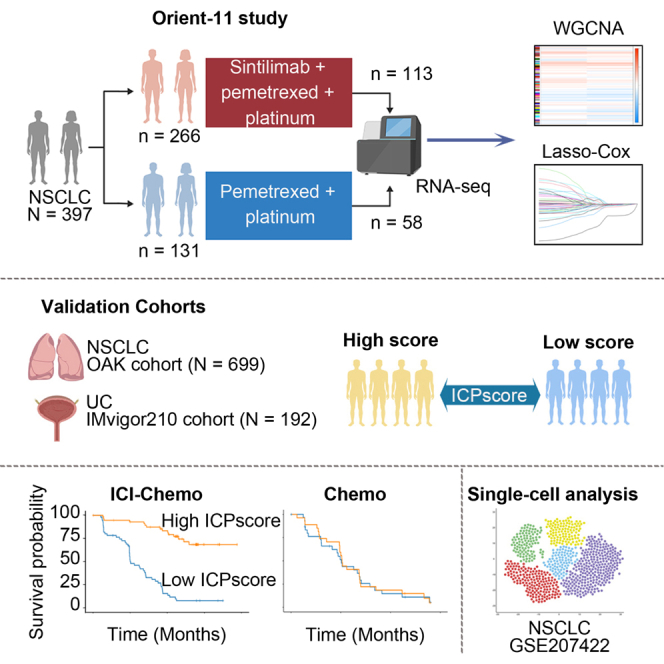

## Introduction

The advent of immune checkpoint inhibitors (ICIs) targeting programmed death protein or ligand (PD-1/PD-L1) axis has redefined first-line therapeutic paradigms for advanced non-small cell lung cancer (NSCLC), with combination regimens integrating ICIs and platinum-based chemotherapy demonstrating unprecedented survival benefits across pivotal trials.^1^^,^^2^^,^^3^^,^^4^^,^^5^

Despite these advances, there are still patients who exhibit primary resistance to ICI plus Chemotherapy (ICI-Chemo), experiencing rapid progression within 6 months while incurring unnecessary toxicity burdens. This clinical dilemma underscores the critical need for predictive biomarkers to optimize patient selection—a challenge exacerbated by the limited utility of established markers such as PD-L1 expression and tumor mutational burden (TMB) in combinatorial settings.^6^^,^^7^^,^^8^

Gene expression signatures in tumor tissue have been explored as potential alternative biomarkers to predict response to ICIs, such as the T cell inflamed signature (TIS),^9^ and Cytolytic Activity (CYT) score.^10^ However, those established signatures were derived from the ICI monotherapy cohort or were only knowledge based, unable to capture ICI-Chemo induced microenvironmental remodeling. This knowledge gap highlights the necessity of developing regimen-specific predictive models grounded in prospective clinical trial data.

Building on the phase III ORIENT-11 trial (NCT03607539) that demonstrated the superiority of sintilimab plus chemotherapy as first-line therapy for advanced NSCLC,^3^ we conducted whole transcriptome sequencing of baseline tumor samples to develop an Immune-Chemotherapy Prediction Score (ICPscore). Through integrative bioinformatics approaches, we identified and validated a robust 9-gene signature that outperforms existing biomarkers in predicting ICI-Chemo responsiveness, with its predictive value further confirmed in the independent OAK trial and IMvigor210 cohort. Subsequent single-cell dataset analysis revealed the 9-gene ICPscore’s association with distinct tumor microenvironment features, while integrated analysis of ORIENT-11, OAK, and The Cancer Genome Atlas (TCGA) datasets elucidated its underlying biological functions. This work provides an evidence-based framework for the precision selection of patients with NSCLC most likely to derive durable benefit from first-line ICI-Chemo combinations.

## Results

### Biomarker-evaluable population characteristics

The ORIENT-11 trial randomized patients to receive sintilimab plus chemotherapy (*n* = 266) or chemotherapy alone (*n* = 131). In this retrospective biomarker analysis, we evaluated a biomarker-evaluable population (BEP) with available baseline PD-L1 tumor proportion score (TPS) and RNA-sequencing data, comprising 113 patients in the sintilimab-chemotherapy arm and 58 in the chemotherapy arm (Figure S1). The baseline characteristics between the BEP (*n* = 171) and biomarker non-evaluable populations (BNEPs, *n* = 226) were balanced, indicating a low risk of substantial selection bias (Table S1). Baseline PD-L1 immunohistochemistry (IHC) and RNA-sequencing were performed on pretreatment tumor specimens. Baseline characteristics and efficacy outcomes were comparable between treatment arms in the BEP (Table S2).

### Identification of immune-checkpoint inhibitor-chemo-related hub genes and construction of immune-chemotherapy prediction score

To identify the hub genes specifically associated with ICI-Chemo efficacy and avoid the effect of subsequent treatments or crossover, we selected the progression-free survival (PFS) as the main clinical trait and performed WGCNA analysis on groups receiving ICI-Chemo. The adjacency matrix was constructed using Pearson correlation and a soft-thresholding power (β = 3) to achieve scale-free topology (Figures S2A–S2B). Through dynamic tree cutting and module merging, 80 distinct gene modules were identified, and their eigengenes were correlated with clinical survival outcomes to assess module-trait relationships (Figures S2C-S2D). According to calculated module-trait relationships, the dark turquoise and gray60 modules showed the strongest correlation with survival benefit (Figures S2E and S2F). Gene Ontology biological processes (GOBP) analysis revealed that the 750 genes within these modules were mostly involved in leukocyte activation, antigen processing/presentation, immune response regulation, and cytokine production regulation (Figure S2G). Through gene significance ranking and log rank filtering (*p* < 0.0001), we identified 45 hub genes predictive of ICI-Chemo response (Table S3).

**Figure 1:**
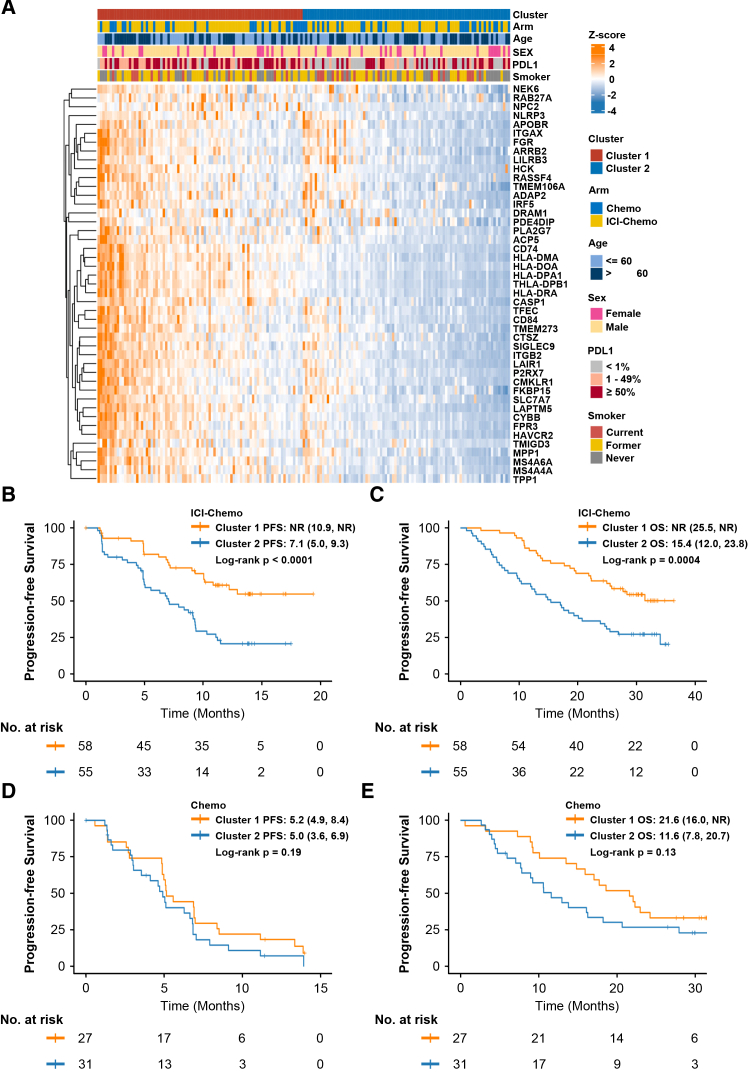
Consensus clustering of 45 hub genes predicts survival outcomes in the ORIENT-11 study (A) Expression pattern of 45 hub genes in the ORIENT-11 cohort (*N* = 171). (B and C) Progression-free survival (PFS) and overall survival (OS) of ICI-chemo based on the consensus clustering of 45 hub genes. (D and E) PFS and OS of chemotherapy based on the consensus clustering of 45 hub genes.

Consensus clustering based on these 45 genes partitioned the cohort (*n* = 171) into two distinct subgroups (Figures S3A–S3D). Figure 1A showed that these hub-genes exhibited different expression patterns in our cohort, while the distribution of clinicopathological characteristics did not vary significantly between the two groups. Cluster 1 demonstrated significantly improved PFS (HR = 0.38, 95% CI: 0.23–0.62, *p* < 0.0001) and overall survival (OS; HR = 0.43, 95% CI: 0.27–0.70, *p* = 0.0004) in the ICI-chemotherapy arm, with no differential benefit observed in the chemotherapy arm (Figures 1B–1E). GSVA enrichment analysis confirmed the enrichment of immune-related pathways in Cluster 1 (Figure S3E).

**Figure 2:**
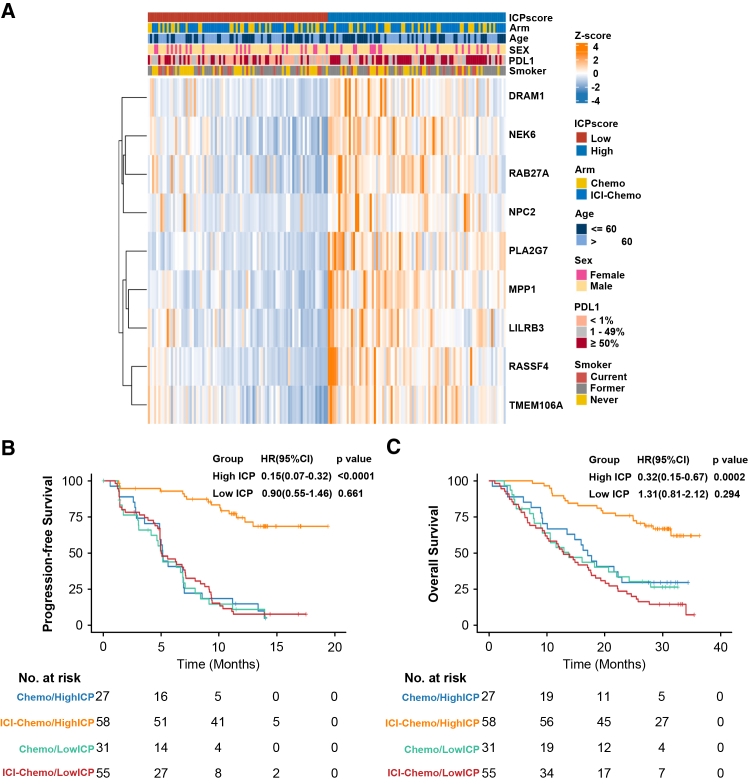
The 9-gene ICPscore stratifies ICI-chemo benefit in the ORIENT-11 Study (A) Expression pattern of 9 genes in the ORIENT-11 cohort (*N* = 171). (B) PFS of ICI-Chemo versus chemotherapy stratified by ICPscore. (C) OS of ICI-Chemo versus chemotherapy stratified by ICPscore.

To optimize clinical applicability, LASSO Cox regression (Figure S4) refined the 45-gene panel to a 9-gene signature (*DRAM1, LILRB3, MPP1, NEK6, NPC2, PLA2G7, RAB27A, RASSF4,* and *TMEM106A*) strongly associated with survival outcomes and exhibited treatment-specific predictive value (Figures S5A–S5D). The 9 genes exhibited distinct expression patterns without significant clinicopathological confounding between the low-score and high-score group (Figure 2A). The ICPscore was subsequently derived using expression levels and LASSO coefficients of these genes (method details). The flowchart of ICPscore construction and annotation for the 9 genes in our model was summarized in Figure S6 and Table S4.

### Immune-chemotherapy prediction score stratifies immune-checkpoint inhibitor plus chemotherapy benefit

Using the median ICPscore as a cutoff, patients were stratified into high- (*n* = 85) and low-score (*n* = 86) subgroups. High ICPscore patients receiving ICI-Chemo demonstrated superior PFS (HR = 0.15, 95% CI: 0.07–0.32, *p* < 0.0001) and OS (HR = 0.32, 95% CI: 0.15–0.67, *p* = 0.0002) compared to chemotherapy, whereas low ICPscore patients showed no significant benefit (PFS: HR = 0.90, 95% CI: 0.55–1.46, *p* = 0.661; OS: HR = 1.31, 95% CI: 0.81–2.21, *p* = 0.294) (Figures 2B and 2C). Tumor response across treatment arms within ICPscore subgroups mirrored these findings (Figures S7A and S7B). Moreover, the multivariate Cox proportional hazard interaction test confirmed significant treatment-by-ICPscore interactions for both PFS (z = −4.70, *p* < 0.0001) and OS (z = −3.42, *p* = 0.0006). Furthermore, to address potential selection bias arising from the BEP being a subset of the overall trial cohort, we performed sensitivity analysis using the inverse probability of treatment weighting (IPTW) and confirmed the robust association between ICPscore and treatment efficacy for both PFS (z = −4.87, *p* < 0.0001) and OS (z = −3.40, *p* = 0.0007) (Table S5).

Notably, ICPscore predicted survival exclusively within the ICI-Chemo cohort (High vs. Low: PFS, HR = 0.15, 95% CI: 0.09–0.26, *p* < 0.0001; OS, HR = 0.22, 95% CI: 0.13–0.36, *p* < 0.0001) with no predictive value in chemotherapy-treated patients (PFS: HR = 0.88, 95% CI: 0.51–1.52, *p* = 0.631; OS: HR = 0.89, 95% CI: 0.48–1.63, *p* = 0.697) (Figures S8A–S8D). Clinicopathologic characteristics of patients in different ICPscore groups are displayed in Table S6, with main characteristics generally balanced between treatment arms across the high ICPscore and low ICPscore groups.

### External validation of immune-chemotherapy prediction score

**Figure 3:**
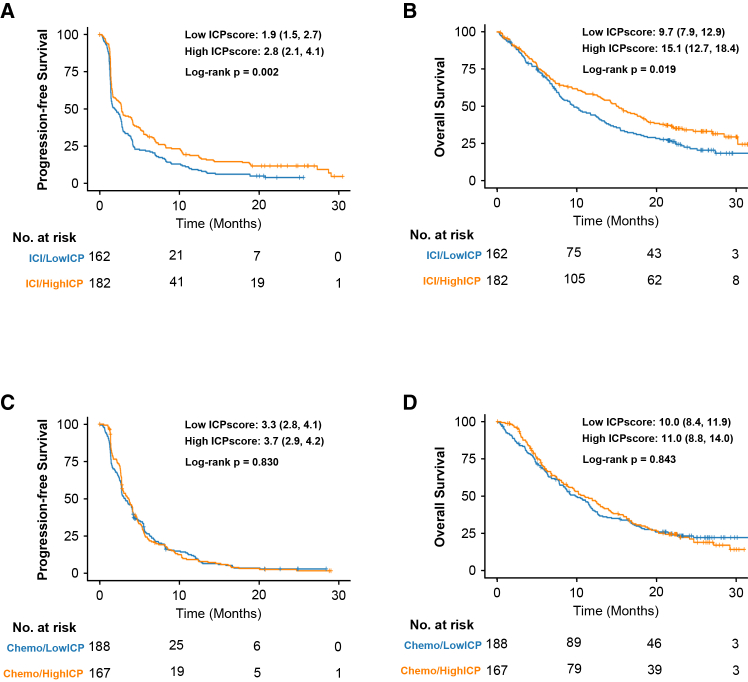
The 9-gene ICPscore stratifies ICI benefit in the OAK study (*N* = 699) (A and B), PFS and OS of atezolizumab based on ICPscore subtypes. (C and D), PFS and OS of chemotherapy based on ICPscore subtypes.

To confirm the predictive value of our ICPscore, we analyzed another independent, phase 3 trial (the OAK study, *n* = 699), which demonstrated similar PFS but significant improvement of OS with atezolizumab compared with chemotherapy in previously treated NSCLC.^11^ We first assessed the association between the ICPscore, treated as a continuous variable, and survival outcomes in the OAK study. We found that a higher ICPscore was significantly associated with improved survival outcomes in the immunotherapy arm (PFS: HR = 0.87, 95% CI: 0.78–0.97, *p* = 0.010; OS: HR = 0.86, 95% CI: 0.76–0.98, *p* = 0.022). Conversely, in the chemotherapy arm of the OAK study, the ICPscore showed no predictive value for PFS (HR = 0.93, 95% CI: 0.83–1.03, *p* = 0.175) or OS (HR = 0.93, 95% CI: 0.82–1.05, *p* = 0.233). We then stratified all patients into high- and low-ICPscore groups based on the median value of ICPscore (349 vs. 350 patients, respectively). Similar findings were observed in OAK study, ICPscore maintained predictive specificity for ICI efficacy (PFS: HR = 0.71, 95% CI: 0.57–0.89, *p* = 0.002; OS: HR = 0.74, 95% CI: 0.57–0.96, *p* = 0.019), with no predictive effect in the docetaxel arm (PFS: HR = 0.98, 95% CI: 0.79–1.21, *p* = 0.830; OS: HR = 0.98, 95% CI: 0.77–1.24, *p* = 0.843) (Figures 3A–3D). High ICPscore patients receiving atezolizumab exhibited significantly improved OS versus chemotherapy (HR = 0.74, 95% CI: 0.57–0.96, *p* = 0.020), whereas low-score patients derived no OS benefit (HR = 0.99, 95% CI: 0.78–1.27, *p* = 0.961). The objective response rate (ORR) was also higher for patients receiving atezolizumab than those receiving chemotherapy in the high ICP score group, yet similar in the low ICPscore group (Figures S9A–S9D). Treatment-by-ICPscore interactions analysis showed that the magnitude of the ICPscore favorable effect was significantly greater in the atezolizumab group than in the chemotherapy group (OS: z = −2.21, *p* = 0.027) (Table S5). Clinicopathologic characteristics were generally balanced between treatment arms across the high ICPscore and low ICPscore groups (Table S7).

To further validate our model’s effectiveness in predicting immunotherapy response, we conducted a supplementary analysis on the IMvigor210CoreBiologies dataset, which is derived from a phase II clinical trial (IMvigor210) evaluating PD-L1 inhibitors (atezolizumab) in cisplatin-ineligible metastatic patients with urothelial cancer.^12^ Consistent with prior findings, the ICPscore, analyzed as a continuous variable, demonstrated a significant association with improved survival in the IMvigor210 cohort (OS: HR = 0.48, 95% CI: 0.25–0.92, *p* = 0.027). When we stratified all patients into high- and low-ICPscore groups based on the median value of ICPscore, patients in the high-score group presented with a significantly longer OS than that of the low-score group (HR = 0.70, 95% CI: 0.50–0.97, *p* = 0.020). Immune phenotyping of bladder cancer in the IMvigor210 dataset included three subtypes: inflamed, excluded, and desert immune. To our surprise, the high-score group had a significantly higher proportion of inflamed tumors than desert tumors (*p* = 0.0002) (Figure S10). To comprehensively assess the predictive stability of the ICPscore, we employed time-dependent receiver operating characteristic (ROC) analysis in different cohorts. The analysis confirmed the ICPscore’s treatment-specific predictive utility. In the ORIENT-11 discovery cohort (ICI-Chemo), the model exhibited strong discriminatory ability, while in immunotherapy-monotherapy validation cohorts (OAK, IMvigor210), it retained moderate performance. Conversely, the ICPscore failed to predict outcomes in chemotherapy-only arms, underscoring its specificity to immunotherapy response. These results highlight the model’s clinical relevance for optimizing patient selection in immunotherapy contexts (Figure S11).

### Immune-chemotherapy prediction score and programmed death protein or ligand 1 expression

**Figure 4:**
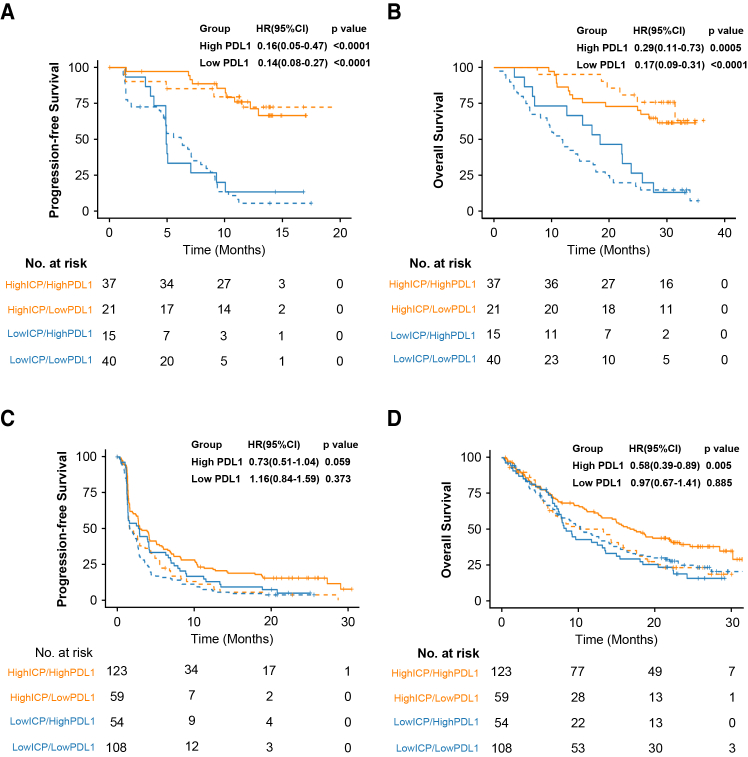
Correlation between ICPscore and survival outcomes following immunotherapy, stratified by PD - L1 expression (A and B) PFS and OS in the ORIENT-11 study for tumors with high and low ICPscores, further classified according to PD - L1 expression levels (above or below the median). (C and D) PFS and OS in the OAK study for tumors with high and low ICPscores, stratified by PD - L1 expression levels (above or below the median). In the figures, the blue line represents the Low ICPscore group, while the yellow line stands for the High ICPscore group. The light line indicates the Low-PD-L1 group, and the bold line represents the High-PD-L1 group.

Previous studies have shown that the detection of PD-L1 protein expression by IHC and quantification of PD-L1 mRNA levels are associated with improved survival benefits of immunotherapy for NSCLC.^13^^,^^14^ In the ORIENT-11 and OAK studies, PD-L1 immunohistochemistry data were available for 100% (171/171) and 51.6% (361/699) of patients, respectively. In both studies, PD-L1 expression (IHC/mRNA) correlated positively with ICPscore (Figures S12A–12D). To explore whether the ICPscore is dependent on PDL1 expression, we further stratified patients based on the ICPscore and PDL1 expression level. As shown in Figure 4, the better survival outcomes in high ICPscore tumor maintained across both high and low PD-L1 mRNA expression subgroups in both ORIENT-11 and OAK study. The ICPscore retained its additive stratification power regardless of the level of PD-L1 expression.

Except for PDL1 expression, it has been reported that some gene expression signatures, such as the T cell-inflamed signature (TIS), cytotoxic activity (CYT),^10^ the interferon (IFN)-γ signature,^9^ and the 4-gene inflammation signature,^15^ could predict tumor response to immunotherapy. Compared to existing signatures, ICPscore showed superior predictive performance for first-line ICI-Chemo benefit despite minimal gene overlap (Table S8; Figures S13A and S13B).

### High immune-chemotherapy prediction score as a biomarker of immune-active tumor microenvironment

**Figure 5:**
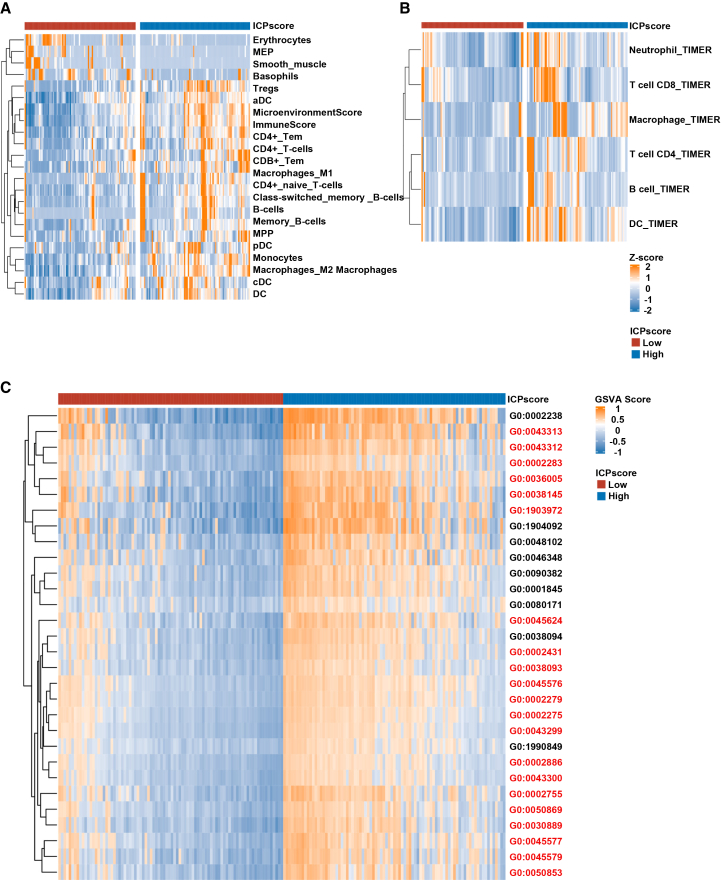
Immunologic features of ICPscore subgroups in the ORIENT-11 study (A and B) High ICPscore subgroups showed significantly enhanced immune cell infiltration, as assessed by Xcell and Timer. (C) Unsupervised clustering revealed enriched immune-related biological functions in the high ICPscore group (highlighted in red).

The association between tumor microenvironment characteristics and survival outcomes in immunotherapy has been extensively explored across multiple clinical trials.^16^^,^^17^ To systematically investigate the relationship between ICPscore and tumor immune microenvironment, we first employed Xcell and TIMER algorithms to profile immune cell infiltration across ICPscore-defined subgroups. Consistently in both the ORIENT-11 and OAK cohorts, tumors with high ICPscore exhibited significantly elevated infiltration of immunologically active components, including CD8^+^ T cells, dendritic cells, and macrophages, compared to their low ICPscore counterparts (Figures 5A, 5B, and S14A and S14B). Complementing these findings, GSVA enrichment analysis demonstrated strong associations between high ICPscore and activation of key immune pathways, such as Toll-like receptor signaling, T cell receptor signaling, chemokine signaling, and leukocyte *trans*-endothelial migration (Figures 5C and S14C). Notably, parallel analyses in the TCGA cohort revealed congruent patterns of immune cell composition and pathway activation between ICPscore subgroups, further validating the robustness of these observations (Figures S15A–S15C).

**Figure 6:**
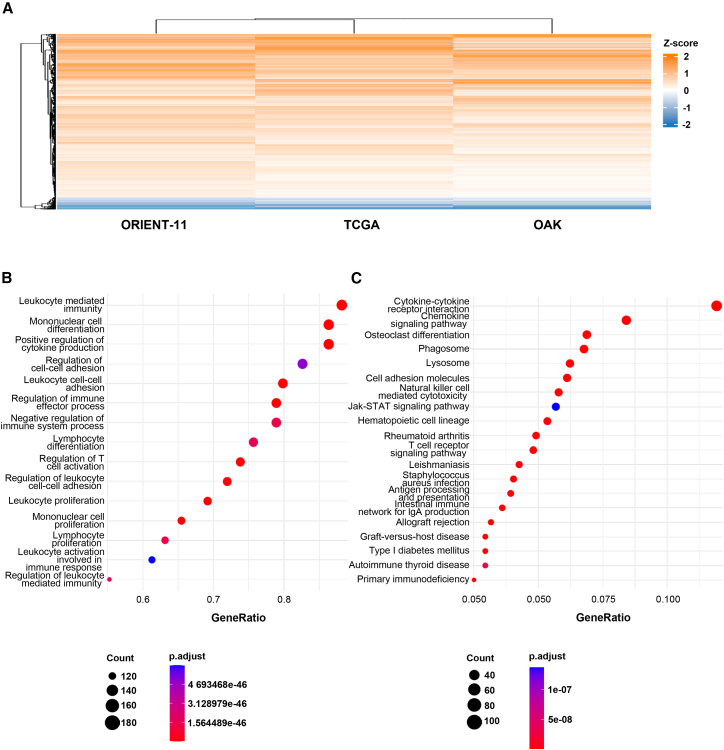
Identification and functional enrichment of common differentially expressed genes (DEGs) across three datasets (A) Heatmap shows the intersection of DEGs from the ORIENT-11, OAK, and TCGA datasets. (B) Bubble plot of Gene Ontology Biological Process (GOBP) enrichment analysis for the common DEGs. (C) Bubble plot of Kyoto Encyclopedia of Genes and Genomes (KEGG) pathway enrichment analysis for the common DEGs. Bubble size represents the number of genes enriched in each term, and color indicates the adjusted *p*-value.

**Figure 7:**
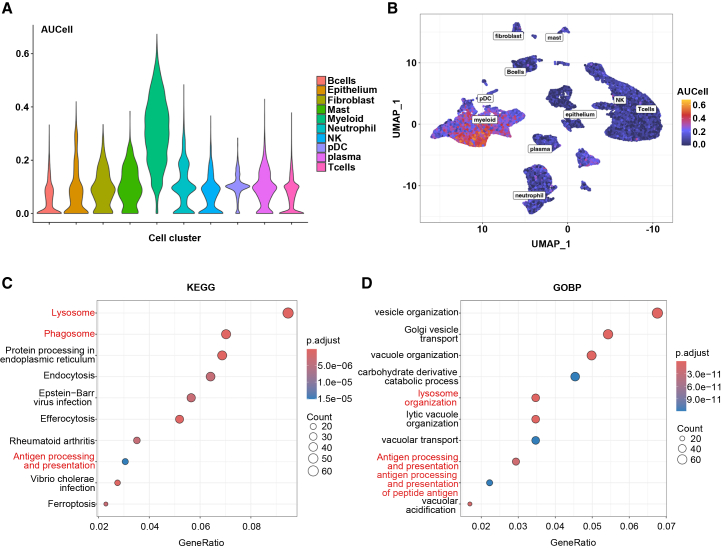
Functional characterization of the 9-gene defined ICPscore in NSCLC tumor microenvironment (A) The ICPscore-related AUCell score of different cell subpopulations in the GSE207422 dataset. (B) UMAP for all single cells; each cell is color-coded based on the ICPscore-related AUCell score. (C) KEGG pathway enrichment analysis (FDR<0.05) of DEGs between high- and low-AUCell score subsets of myeloid cells. (D) GOBP pathway enrichment analysis (FDR<0.05) of DEGs between high- and low-AUCell score subsets of myeloid cells.

To comprehensively characterize the transcriptional landscape of ICPscore-stratified tumor microenvironments, we integrated differentially expressed genes (DEGs) from the ORIENT-11, OAK, and TCGA cohorts. Strikingly, 2634 DEGs showed consistent directional changes across all three datasets (Figure 6A). Functional enrichment analysis of these shared DEGs revealed predominant involvement in immune regulatory pathways, including cytokine-cytokine receptor interactions, antigen processing/presentation, NK cell-mediated cytotoxicity, and multiple receptor signaling pathways (T cell, B cell, and NOD-like receptors) (Figures 6B, 6C, S16A, and S16B). Complete gene lists are cataloged in Table S9. To elucidate the role of the 9-gene ICPscore in shaping the tumor immune microenvironment of NSCLC, we leveraged the publicly available NSCLC single-cell RNA-seq dataset GSE207422.^18^ Cells were clustered into 10 major clusters after quality control (Figures S17A and S17B). We next calculated the relative expression of the 9 genes for each cell using the AUCell method, which quantifies gene set activity in single-cell RNA-seq data by ranking gene expression within each cell. We found a pronounced enrichment of the ICPscore-based AUCell score within myeloid lineages, prompting further functional stratification (Figures 7A and 7B). The isolated myeloid cell subset was then stratified into high- and low-score groups using the top and bottom quartiles of the AUCell score distribution within this population (Figure S17C). Differential expression analysis between these high and low-score myeloid subgroups revealed that cells with a high AUCell score exhibited marked upregulation of immune-effector pathways, including antigen processing/presentation, phagosome, and lysosome, as evidenced by Kyoto Encyclopedia of Genes and Genomes (KEGG) pathways and GOBP enrichment (Figures 7C and 7D). These findings position the ICPscore as a biomarker of myeloid functional activation. Collectively, these multi-cohort analyses establish that the 9-gene ICPscore signature effectively identifies tumors with immunologically active microenvironments. This mechanistic insight aligns with clinical observations, suggesting that patients exhibiting elevated ICPscore may derive enhanced therapeutic benefit from immune checkpoint blockade therapies.

## Discussion

Despite advancements in ICI-based therapies, the lack of robust biomarkers to identify patients benefiting from ICI chemo combinations remains a critical unmet need in advanced NSCLC management.^19^ While existing biomarkers such as PD-L1 demonstrate limited predictive value in combinatorial settings,^4^^,^^5^^,^^7^ our study establishes a dedicated 9-gene ICPscore through systematic analysis of phase III trial cohorts, offering superior predictive capability for treatment stratification.

By integrating transcriptomic data from the ORIENT-11 discovery cohort (*N* = 171), we demonstrate that high ICPscore patients derived significant survival benefits from first-line ICI-Chemo, whereas low ICPscore patients achieved comparable outcomes with chemotherapy alone. We further validated the predictive value of ICPscore in the independent OAK trial (*N* = 699) and IMvigor210 study (*N* = 192). The consistent performance of ICPscore across distinct cohorts-ORIENT-11 and OAK/IMvigor210 (ICI monotherapy)-suggests that ICPscore may function as a general biomarker for ICI benefit, reflecting key tumor microenvironment features that are essential for immunotherapy response. Meanwhile, this cross-context validity raises important clinical implications. For patients with high ICPscore, our data suggest that the observed benefit is primarily driven by the ICI component. This raises the provocative possibility of chemotherapy de-escalation in such patients, which could reduce toxicity while maintaining efficacy—a hypothesis that warrants prospective validation in tailored trials comparing ICI-chemotherapy versus ICI monotherapy in high-scoring patients. Conversely, for patients with low ICPscore who derived no significant benefit from ICI-Chemo compared with chemotherapy, our findings strongly support the exploration of alternative strategies, including chemotherapy alone or combinations incorporating anti-angiogenic agents.^20^

Our analytical approach leveraged WGCNA to identify therapy-responsive hub genes from which a clinically implementable 9-gene panel was distilled using LASSO regression.^21^^,^^22^ This methodology overcomes limitations of previous signatures derived from ICI monotherapy cohorts, which often focus predominantly on T cell metrics and may fail to capture the broader immune-priming effects of chemotherapy. Notably, although PFS was selected for the discovery phase to reduce potential confounding from subsequent therapies, the derived ICPscore showed strong predictive ability for OS in independent validation cohorts. This highlights the score’s robustness, confirming its value in forecasting long - term clinical benefits and increasing its clinical relevance.

While PD-L1 expression correlated with ICPscore, our analysis confirmed the latter’s superior predictive value. This advantage likely stems from ICPscore’s multi-dimensional characterization of tumor-immune interactions, capturing biological features beyond the inflammatory status reflected by PD-L1. Mechanistic analyses revealed that ICPscore is associated with both adaptive immune processes, such as MHC-I presentation (via LILRB3) and lymphocyte exocytosis (via RAB27A), and innate immune modulation, including neutrophil polarization (via MPP1) and macrophage regulation (via TMEM106A). Notably, the inclusion of metabolic regulators related to autophagy (DRAM1) and cholesterol metabolism (NPC2) expands the current understanding of ICI-chemotherapy synergism. These findings align with emerging evidence that chemotherapy-induced immunogenic cell death creates permissive microenvironments for ICIs,^23^^,^^24^ while providing transcriptomic evidence that baseline lysosomal/metabolic states determine therapeutic vulnerability to such combinations.

Cross-cohort (ORIENT-11, OAK, and TCGA) validation revealed that a high ICPscore is associated with activated anti-tumor pathways, including cytokine-chemokine signaling, antigen processing and presentation, and lymphocyte cytotoxicity—biological hallmarks of immunologically “hot” tumors.^25^^,^^26^ Our single-cell analysis established the 9-gene ICPscore as a signature of myeloid cell activation in NSCLC. This activation is driven by the coordinated upregulation of antigen processing/presentation and lysosomal pathways, which functionally enhances the capacity for tumor antigen surveillance and innate-adaptive immune crosstalk, thereby providing a mechanistic basis for its predictive utility.

It is critical to acknowledge the substantial distinctions between the training and validation cohorts when interpreting the attenuated performance of the ICPscore in the OAK trial. The OAK cohort featured a predominantly White population receiving second-line atezolizumab monotherapy, whereas ORIENT-11 studied an Asian NSCLC population on first-line sintilimab-chemotherapy. These differences in therapy line, specific agent, and treatment regimen likely contribute to this performance drop. Notably, the score’s limited efficacy (AUC ≈0.5) in chemotherapy-only cohorts confirms its specificity for predicting immunotherapy response, thereby reinforcing its biological relevance. These findings collectively argue for prudent interpretation and further validation prior to its widespread clinical implementation.

In conclusion, the ICPscore is a validated transcriptomic biomarker specifically developed for ICI-Chemo response prediction in NSCLC. Its application could facilitate biomarker-driven stratification, helping to prevent unnecessary toxicity and financial burden in potential non-responders while optimizing resource allocation for precision immunotherapy. Future studies should investigate the utility of ICPscore in guiding sequential therapy and explore its biological underpinnings through functional genomics approaches.

### Limitations of the study

First, the retrospective design and moderate sample size necessitate prospective validation in larger, independent cohorts and across diverse tumor types. Second, while RNA-based assays show clinical feasibility,^27^ technical standardization across platforms remains a critical challenge and requires further optimization. Nevertheless, the use of data from two randomized phase III trials strengthens the evidence by minimizing selection biases inherent in observational studies.

## Resource availability

### Lead contact

Further information and requests for resources should be directed to and will be fulfilled by the lead contact, Wenfeng Fang (fangwf@sysucc.org.cn).

### Materials availability

This study did not generate new unique reagents.

### Data and code availability

Gene expression data for the TCGA lung adenocarcinoma (LUAD) and squamous cell carcinoma (LUSC) are publicly available. RNA-seq data and corresponding clinical data for OAK and IMvigor210 are available in the European Genome-phenome Archive (EGA) (https://ega-archive.org/) with restricted access (EGA: EGAS00001004343 and EGAS00001005013). Lung cancer scRNA-seq data (GSE207422) were downloaded from the Gene Expression Omnibus (GEO) database. Due to privacy and ethical concerns, the RNA sequencing data for ORIENT-11 must be managed under controlled access. Data is hosted at the OMIX repository of the China National Center for Bioinformation (NGDC, https://ngdc.cncb.ac.cn/omix), and the accession number is OMIX012889. Requests to access data should follow the specific guidance available at the NGDC database portal (https://ngdc.cncb.ac.cn). The data access committee, guided by the DAC chair (Li Zhang, zhangli@sysucc.org.cn) regulates access in accordance with institutional and national guidelines. Data are permitted for non-commercial academic research only. The user can also contact the corresponding author directly for enquiries. To ensure full reproducibility, we have provided complete methodological details and the model algorithm in the method details section, enabling independent validation. These accession numbers for the datasets are listed in the key resources table.

This article does not report original code. Any additional information required to reanalyze the data reported in this article is available from the lead contact upon request.

## Acknowledgments

The study was funded by the Chinese National Natural Science Foundation Project (grant numbers: 82373262, 82173101, 82272789). This study was conducted in accordance with the Declaration of Helsinki and was approved by Sun Yat-sen University Cancer Center Institutional Review Board (B2023-468-01). Written informed consent was obtained from all patients. The graphical illustrations in this article were created with BioGDP.com (https://www.biogdp.com).^28^

## Author contributions

Conceptualization, W. F.; data curation, J. L.; formal analysis, J. L. and J. H.; funding acquisition, W. F. and L. Z.; methodology, L. P.; writing-original draft, J. L.; writing-review and editing, X. C. and W. Z.; investigation, X. C., S. H., G. C., Y. Z., and T. Z.; resources, W. F., L. Z., and Y. Y.; study supervision, W. F. All authors: Final approval of the article.

## Declaration of interests

The authors declare no competing interests.

## STAR★Methods

### Key resources table

**Table:** 

REAGENT or RESOURCE	SOURCE	IDENTIFIER
**Biological samples**		

Bulk tissues from NSCLC patients in ORIENT-11 clinical trial	Yang et al.^2^	OMIX012889

**Critical commercial assays**		

PD-L1 IHC 22C3 pharmDx	Agilent Technologies	Cat#SK006; RRID: AB_2889976

**Deposited data**		

Bulk tissues from NSCLC patients in ORIENT-11 clinical trial	Yang et al.^2^	OMIX012889
Bulk RNA-seq data from OAK clinical trial	Rittmeyer et al.^11^	EGAS00001005013
Bulk RNA-seq data from IMvigor210 clinical trial	Powles et al.^12^	EGAS00001004343
Single-cell RNA sequencing of tumor from NSCLC patients	Hu et al.^18^	GSE207422
TCGA-LUSC RNA-seq data	The Cancer Genome Atlas	N/A
TCGA-LUAD RNA-seq data	The Cancer Genome Atlas	N/A

**Software and algorithms**		

R 4.2.1	R Development Core Team	https://cran.r-project.org/
WCGNA	Langfelder et al.^29^	https://cran.r-project.org/web/packages/WGCNA/index.html
ClusterProfiler	Yu et al.^30^	https://bioconductor.org/packages/release/bioc/html/clusterProfiler.html
ConsensusClusterPlus	Wilkerson et al.^31^	https://bioconductor.org/packages/release/bioc/html/ConsensusClusterPlus.html
pheatmap	Raivo^32^	https://cran.r-project.org/package=pheatmap
TIMER	Li et al.^33^	https://cistrome.shinyapps.io/timer/
xCell	Aran et al.^34^	https://github.com/dviraran/xCell
Gene Set Variation Analysis (GSVA)	Hänzelmann et al.^35^	https://bioconductor.org/packages/release/bioc/html/GSVA.html
limma	Ritchie et al.^36^	https://bioconductor.org/packages/release/bioc/html/limma.html
DESeq2	Love et al.^37^	https://bioconductor.org/packages/release/bioc/html/DESeq2.html
Seurat	Hao et al.^38^	https://cran.r-project.org/package=Seurat
AUCell	Aibar et al.^39^	https://bioconductor.org/packages/release/bioc/html/AUCell.html
TimeROC	Blanche et al.^40^	https://cran.r-project.org/package=timeROC

### Experimental model and study participant details

#### Study design and participants

ORIENT-11 is a randomized, double-blind, phase 3 study conducted in 47 centers in the People’s Republic of China (ClinicalTrials.gov identifier: NCT03607539). The design and patient eligibility criteria of ORIENT-11 trial has been described in detail previously.^2^^,^^3^ Briefly, patients with previously untreated, locally advanced, or metastatic nonsquamous NSCLC without sensitizing EGFR or anaplastic lymphoma kinase (ALK) genomic aberration were randomized (2:1 ratio) to receive either sintilimab (*n* = 266) or placebo plus pemetrexed and platinum (*n* = 131) once every 3 weeks for four cycles, followed by sintilimab or placebo plus pemetrexed as maintenance therapy. The treatment was continued until disease progression, intolerable toxicity, initiation of a new treatment, or withdrawal of consent. The primary endpoint was PFS assessed by a blinded independent radiographic review committee. OS and ORR were secondary endpoints. Tissue samples at baseline were required for PD-L1 expression assessment and transcriptome sequencing. The clinical protocol was approved by the respective institutional review boards and ethics committees. All participants provided written informed consent. Baseline clinical and pathological characteristics of patients in the ORIENT-11 cohorts are presented in Tables S1 and S2.

### Method details

#### RNA sequencing and data processing

In the ORIENT-11 trial, tumor specimens were collected via core biopsy. RNA extraction from formalin-fixed, paraffin-embedded baseline tumor samples was performed using the RNeasy FFPE Kit (Qiagen), and ribosomal RNAs were removed using the NEBNext rRNA Depletion Kit (NEB). The NEBNext Ultra II Directional RNA Library Prep Kit for Illumina was employed for cDNA library preparation, following the manufacturer’s protocol. Fragmentation was first carried out using divalent cations, followed by strand-specific construction of RNA-sequencing libraries. Sequencing was conducted on the NovaSeq 6000 platform (Illumina). Quality control of reads was assessed using FastQC, and raw reads were filtered with Trimmomatic before being aligned to the human GRCh38 reference genome using the STAR aligner.^41^ For downstream analysis, only samples with a unique reads mapping rate of at least 30% and a reads duplication rate of no more than 90% were selected. The HTSeq-count tool was utilized to count reads mapped to each gene.^42^ Following quantification, the raw gene count matrix underwent initial filtering to exclude lowly expressed or non-informative genes, retaining only those with detectable expression to reduce noise. The filtered counts were normalized using Relative Log Expression (RLE) to correct for library size and compositional biases. ComBat was applied to log-transformed data to mitigate technical variation (batch effects). Finally, normalized counts were converted to Transcripts Per Million (TPM), incorporating gene length adjustments to enable cross-sample comparisons. The resulting matrix was ready for downstream analyses like visualization and differential expression testing.

#### PD-L1 immunohistochemistry assessment

The PD-L1 immunohistochemistry (IHC) was performed on formalin-fixed, paraffin-embedded (FFPE) tumor tissue collected at baseline. PD-L1 expression was assessed by the 22C3 pharmDx assay (Agilent Technologies) at a central laboratory (Covance, Shanghai, People’s Republic of China). Tumor PD-L1 expression level was measured by tumor proportion score (TPS), defined as the percentage of viable tumor cells revealing partial or complete membrane staining at any intensity.

#### Identification of gene modules related to ICI-Chemo efficacy

Weighted Gene Coexpression Network Analysis (WGCNA)^29^ was performed to identify co-expressed gene modules and explore their associations with ICI-Chemo efficacy. Prior to network construction, genes with invariant expression (standard deviation <1 across all samples) or missing values in >5% of samples were filtered out, retaining 29204 genes for downstream analysis. The adjacency matrix was calculated based on pairwise Pearson correlation coefficients, and a soft-thresholding power (β) of 3 was selected to ensure a scale-free network. Gene modules were identified using dynamic tree cutting with a minimum module size of 50 genes. Modules with highly similar expression profiles (correlation >0.75) were merged using a height cutoff of 0.25. This yielded 80 distinct modules, each represented by its module eigengene (ME). To evaluate module-trait relationships, we calculated the correlation between MEs and clinical survival outcomes. Modules with a correlation coefficient |r| > 0.25 and *p*-value <0.05 were considered significant, including the “darkturquoise” (r = 0.43, *p* = 2.4 × 10^−14^) and “grey60” (r = 0.36, p = *p* = 1.3 × 10^−15^) modules, suggesting their potential roles in ICI-Chemo response. Hub genes within significant modules were identified based on gene significance (GS > 0.1, p.GS < 0.05). Functional enrichment analyses were performed on genes within significant modules using the clusterProfiler R package (Figure S2).^30^

#### Consensus clustering for sample stratification

Samples were grouped into two clusters by consensus clustering analysis with the R package “ConsensusClusterPlus”.^31^ Clustering robustness was enhanced by integrating 1000 iterations of subsampling and clustering. Key parameters included subsampling 80% of samples (pItem = 0.8) and retaining all features (pFeature = 1) in each iteration to ensure broad data representation, while hierarchical clustering with Pearson correlation distance (distance = “pearson”) was employed to capture nonlinear relationships among samples. The optimal cluster number (k = 2) was determined by evaluating consensus cumulative distribution functions (CDFs) and the proportion of ambiguous clustering (PAC) score, where minimal PAC values indicated stable clustering patterns. The t-distributed stochastic neighbor embedding (t-SNE) analysis was performed to validate the stability of the clustering. Gene expression patterns of the stratified patients were visualized using a heatmap generated with the R package “pheatmap”.^32^

#### Construction of immune checkpoint predictive score (ICPscore)

Lasso Cox regression was used to identify the most relevant predictive genes. The optimal value of the Lasso penalty parameter (λ = 0.06235178) was determined by 10-fold cross-validation to minimize the partial likelihood deviance. Using the “glmnet” and “survival” packages in R, we performed the analysis, which included constructing the Lasso Cox model and visualizing the coefficient paths to observe how gene coefficients varied with increasing λ values.^43^ Nine genes whose expression levels were significantly associated with survival outcomes were identified. Each gene’s contribution to the model was quantified by its regression coefficient, reflecting its relative importance in predicting survival.

The ICPscore was calculated as: ICPscore = Σ [Gene Expression × Coefficient]

Coefficients: *DRAM1* (−0.00109), *LILRB3* (−0.00513), *MPP1* (−0.03406), *NEK6* (−0.00264), *NPC2* (−0.00122), *PLA2G7* (−0.00863), *RAB27A* (−0.00457), *RASSF4* (−0.00200), *TMEM106A* (−0.00407).

#### External validation cohorts

The OAK study is a phase 3 randomized controlled trial (NCT02008227) which involved 699 patients with NSCLC refractory to first-line platinum-based chemotherapy. All patients in OAK study received atezolizumab or docetaxel. Eventually, 344 patients treated with atezolizumab and 355 patients treated with docetaxel were included in the current analysis. The OAK trials were done in accordance with the Declaration of Helsinki. The protocol was approved by the local ethics committee, and all participants provided written informed consent. The PD-L1 expression level was analyzes using pharmDx 22C3 assay and scored as the sum of PD-L1–positive tumor cells as a proportion of the total number of viable tumor cells. Detailed methods have been published previously.^11^ The IMvigor210 trial is a multicenter, single-arm, Phase 2 study investigating the efficacy of atezolizumab in patients with metastatic urothelial carcinoma following progression on platinum-based chemotherapy. Both RNA seq and efficacy data from the two cohorts were formally requested from Genentech/Roche at the European Genome-phenome Archive. These datasets were subsequently utilized to investigate the correlation between ICPscore and survival outcome in patients undergoing immunotherapy. ICPscore groups were defined by the median cutoff across the entire cohort, ensuring equal distribution prior to treatment-specific analyses.

#### Immune microenvironment characterization

Transcriptomic data (TPM and counts) for each gene for TCGA cohort (TCGA-LUSC and TCGA-LUAD) were obtained using the TCGAbiolinks R package.^44^^,^^45^^,^^46^ For the ORIENT-11, OAK, and TCGA cohorts, immune cell infiltration was estimated using xCell and TIMER algorithms.^33^^,^^34^ Gene Set Variation Analysis (GSVA) analysis was conducted using the TPM-normalized data by the clusterProfiler package.^30^^,^^35^ The top 30 enriched functional terms of GSVA analysis were selected and visualized for result presentation. All gene sets were downloaded from the MSigDB database.^47^

#### Differential gene expression analysis between high- and low-ICPscore groups

Differentially expressed genes (DEGs) between high- and low-ICPscore groups were identified in the ORIENT-11, OAK, and TCGA cohorts. For RNA-seq count data, DESeq2 was employed, while for TPM data, the limma-voom pipeline was applied.^36^^,^^37^ Enrichment analyses of Kyoto Encyclopedia of Genes and Genomes (KEGG) pathways and Gene Ontology biological processes (GOBP) were subsequently performed on the TPM-normalized data using the clusterProfiler package.^30^^,^^48^ Significant terms and pathways were defined using a stringent dual-threshold of false discovery rate (FDR) < 0.05 and *p*-value <0.05.

#### Single-cell sequencing data analysis

Lung cancer scRNA-seq data (GSE207422) were downloaded from the Gene Expression Omnibus (GEO) database. Raw unique molecular identifier (UMI) matrices were processed using the Seurat R package, with stringent quality control: cells expressing <500 genes, >15% mitochondrial UMIs, or >50% ribosomal UMIs were excluded.^38^ Potential doublets were removed using Scrublet,^49^ yielding a final dataset of 70,065 high-quality cells. Dimensionality reduction was performed via principal component analysis (PCA) on the integration-transformed expression matrix (first 50 PCs), followed by uniform manifold approximation and projection (UMAP) for visualization. Clustering (resolution = 0.5) and cell-type annotation were based on canonical marker genes identified using FindAllMarkers (min.pct = 0.25).^18^ To investigate the biological indication of our 9-gene based ICPscore, we calculated its relative expression per cell using AUCell.^39^ Differential expression analysis comparing cells from high- and low- AUCell score groups was performed using the FindMarkers function with the parameter “min.pct = 0.25, thresh.use = 0.25”.

### Quantification and statistical analysis

Survival outcomes were analyzed by Kaplan-Meier curves with stratified log rank tests. The hazard ratio (HR) and associated 95% confidence intervals (CIs) were derived from Cox proportional-hazards models. Categorical and continuous variables were compared using the chi-square test and Wilcoxon rank-sum/Kruskal-Wallis tests, respectively. A sensitivity analysis using IPTW was conducted to address selection bias.^50^ Weights derived from a propensity score model (incorporating key baseline variables) were applied to Cox regression models for PFS and OS to better approximate the intention-to-treat population. The predictive accuracy of ICPscore was evaluated using time-dependent ROC analysis with the timeROC package, with the area under the curve (AUC) serving as the key metric.^40^ Two-sided *p* < 0.05 indicated significance. Analyses used R v4.2.1.

### Additional resources

Clinical trial registry number of ORIENT-11: NCT03607539, https://clinicaltrials.gov/ct2/show/NCT03607539.

## Supplemental information

Document S1. Figures S1–S17 and Tables S1, S2, and S4–S8

Table S3. Hub genes predictive of ICI-Chemo response, related to Figure 1

Table S9. The differentially expressed genes between high ICPscore group and low ICPscore group from the ORIENT-11, OAK, and TCGA cohorts, related to Figure 6

## References

[1] Wu K. (2022). Research highlights of clinical oncology early 2022. Holist. Integr. Oncol.

[2] Yang Y., Sun J., Wang Z., Fang J., Yu Q., Han B., Cang S., Chen G., Mei X., Yang Z. (2021). Updated Overall Survival Data and Predictive Biomarkers of Sintilimab Plus Pemetrexed and Platinum as First-Line Treatment for Locally Advanced or Metastatic Nonsquamous NSCLC in the Phase 3 ORIENT-11 Study. J. Thorac. Oncol..

[3] Yang Y., Wang Z., Fang J., Yu Q., Han B., Cang S., Chen G., Mei X., Yang Z., Ma R. (2020). Efficacy and Safety of Sintilimab Plus Pemetrexed and Platinum as First-Line Treatment for Locally Advanced or Metastatic Nonsquamous NSCLC: a Randomized, Double-Blind, Phase 3 Study (Oncology pRogram by InnovENT anti-PD-1-11). J. Thorac. Oncol..

[4] Paz-Ares L., Luft A., Vicente D., Tafreshi A., Gümüş M., Mazières J., Hermes B., Çay Şenler F., Csőszi T., Fülöp A. (2018). Pembrolizumab plus Chemotherapy for Squamous Non-Small-Cell Lung Cancer. N. Engl. J. Med..

[5] Gandhi L., Rodríguez-Abreu D., Gadgeel S., Esteban E., Felip E., De Angelis F., Domine M., Clingan P., Hochmair M.J., Powell S.F. (2018). Pembrolizumab plus Chemotherapy in Metastatic Non-Small-Cell Lung Cancer. N. Engl. J. Med..

[6] Duan J., Zhang Y., Chen R., Liang L., Huo Y., Lu S., Zhao J., Hu C., Sun Y., Yang K. (2023). Tumor-immune microenvironment and NRF2 associate with clinical efficacy of PD-1 blockade combined with chemotherapy in lung squamous cell carcinoma. Cell Rep. Med..

[7] Paz-Ares L., Langer C.J., Novello S., Halmos B., Cheng Y., Gadgeel S.M., Hui R., Sugawara S., Borghaei H., Cristescu R. (2019). LBA80Pembrolizumab (pembro) plus platinum-based chemotherapy (chemo) for metastatic NSCLC: Tissue TMB (tTMB) and outcomes in KEYNOTE-021, 189, and 407. Ann. Oncol..

[8] Garassino M., Rodriguez-Abreu D., Gadgeel S., Esteban E., Felip E., Speranza G., Reck M., Hui R., Boyer M., Cristescu R. (2019). OA04.06 Evaluation of TMB in KEYNOTE-189: Pembrolizumab Plus Chemotherapy vs Placebo Plus Chemotherapy for Nonsquamous NSCLC. J. Thorac. Oncol..

[9] Ayers M., Lunceford J., Nebozhyn M., Murphy E., Loboda A., Kaufman D.R., Albright A., Cheng J.D., Kang S.P., Shankaran V. (2017). IFN-γ-related mRNA profile predicts clinical response to PD-1 blockade. J. Clin. Investig..

[10] Rooney M.S., Shukla S.A., Wu C.J., Getz G., Hacohen N. (2015). Molecular and genetic properties of tumors associated with local immune cytolytic activity. Cell.

[11] Rittmeyer A., Barlesi F., Waterkamp D., Park K., Ciardiello F., von Pawel J., Gadgeel S.M., Hida T., Kowalski D.M., Dols M.C. (2017). Atezolizumab versus docetaxel in patients with previously treated non-small-cell lung cancer (OAK): a phase 3, open-label, multicentre randomised controlled trial. Lancet Lond. Engl..

[12] Powles T., Durán I., van der Heijden M.S., Loriot Y., Vogelzang N.J., De Giorgi U., Oudard S., Retz M.M., Castellano D., Bamias A. (2018). Atezolizumab versus chemotherapy in patients with platinum-treated locally advanced or metastatic urothelial carcinoma (IMvigor211): a multicentre, open-label, phase 3 randomised controlled trial. Lancet Lond. Engl..

[13] Daud A.I., Wolchok J.D., Robert C., Hwu W.-J., Weber J.S., Ribas A., Hodi F.S., Joshua A.M., Kefford R., Hersey P. (2016). Programmed Death-Ligand 1 Expression and Response to the Anti-Programmed Death 1 Antibody Pembrolizumab in Melanoma. J. Clin. Oncol..

[14] Fernandez A.I., Gavrielatou N., McCann L., Shafi S., Moutafi M.K., Martinez-Morilla S., Vathiotis I.A., Aung T.N., Yaghoobi V., Bai Y. (2022). Programmed Death-Ligand 1 and Programmed Death-Ligand 2 mRNAs Measured Using Closed-System Quantitative Real-Time Polymerase Chain Reaction Are Associated With Outcome and High Negative Predictive Value in Immunotherapy-Treated NSCLC. J. Thorac. Oncol..

[15] Sangro B., Melero I., Wadhawan S., Finn R.S., Abou-Alfa G.K., Cheng A.-L., Yau T., Furuse J., Park J.-W., Boyd Z. (2020). Association of inflammatory biomarkers with clinical outcomes in nivolumab-treated patients with advanced hepatocellular carcinoma. J. Hepatol..

[16] Hamada T., Soong T.R., Masugi Y., Kosumi K., Nowak J.A., da Silva A., Mu X.J., Twombly T.S., Koh H., Yang J. (2018). TIME (Tumor Immunity in the MicroEnvironment) classification based on tumor CD274 (PD-L1) expression status and tumor-infiltrating lymphocytes in colorectal carcinomas. OncoImmunology.

[17] Shirasawa M., Yoshida T., Shimoda Y., Takayanagi D., Shiraishi K., Kubo T., Mitani S., Matsumoto Y., Masuda K., Shinno Y. (2021). Differential Immune-Related Microenvironment Determines Programmed Cell Death Protein-1/Programmed Death-Ligand 1 Blockade Efficacy in Patients With Advanced NSCLC. J. Thorac. Oncol..

[18] Hu J., Zhang L., Xia H., Yan Y., Zhu X., Sun F., Sun L., Li S., Li D., Wang J. (2023). Tumor microenvironment remodeling after neoadjuvant immunotherapy in non-small cell lung cancer revealed by single-cell RNA sequencing. Genome Med..

[19] Hegde P.S., Chen D.S. (2020). Top 10 Challenges in Cancer Immunotherapy. Immunity.

[20] Shiraishi Y., Kishimoto J., Sugawara S., Mizutani H., Daga H., Azuma K., Matsumoto H., Hataji O., Nishino K., Mori M. (2024). Atezolizumab and Platinum Plus Pemetrexed With or Without Bevacizumab for Metastatic Nonsquamous Non-Small Cell Lung Cancer: A Phase 3 Randomized Clinical Trial. JAMA Oncol..

[21] Wang S., Fan G., Li L., He Y., Lou N., Xie T., Dai L., Gao R., Yang M., Shi Y., Han X. (2023). Integrative analyses of bulk and single-cell RNA-seq identified cancer-associated fibroblasts-related signature as a prognostic factor for immunotherapy in NSCLC. Cancer Immunol. Immunother..

[22] Zhang X., Gao G., Zhang Q., Zhao S., Li X., Cao W., Luo H., Zhou C. (2024). In-depth proteomic analysis identifies key gene signatures predicting therapeutic efficacy of anti-PD-1/PD-L1 monotherapy in non-small cell lung cancer. Transl. Lung Cancer Res..

[23] Hato S.V., Khong A., de Vries I.J.M., Lesterhuis W.J. (2014). Molecular pathways: the immunogenic effects of platinum-based chemotherapeutics. Clin. Cancer Res..

[24] Bracci L., Schiavoni G., Sistigu A., Belardelli F. (2014). Immune-based mechanisms of cytotoxic chemotherapy: implications for the design of novel and rationale-based combined treatments against cancer. Cell Death Differ..

[25] Sharma P., Allison J.P. (2015). Immune checkpoint targeting in cancer therapy: toward combination strategies with curative potential. Cell.

[26] Fridman W.H., Pagès F., Sautès-Fridman C., Galon J. (2012). The immune contexture in human tumours: impact on clinical outcome. Nat. Rev. Cancer.

[27] Gutierrez M., Lam W.-S., Hellmann M.D., Gubens M.A., Aggarwal C., Tan D.S.W., Felip E., Chiu J.W.Y., Lee J.-S., Yang J.C.-H. (2023). Biomarker-directed, pembrolizumab-based combination therapy in non-small cell lung cancer: phase 2 KEYNOTE-495/KeyImPaCT trial interim results. Nat. Med..

[28] Jiang S., Li H., Zhang L., Mu W., Zhang Y., Chen T., Wu J., Tang H., Zheng S., Liu Y. (2025). Generic Diagramming Platform (GDP): a comprehensive database of high-quality biomedical graphics. Nucleic Acids Res..

[29] Langfelder P., Horvath S. (2008). WGCNA: an R package for weighted correlation network analysis. BMC Bioinf..

[30] Yu G., Wang L.-G., Han Y., He Q.-Y. (2012). clusterProfiler: an R package for comparing biological themes among gene clusters. OMICS A J. Integr. Biol..

[31] Wilkerson M.D., Hayes D.N. (2010). ConsensusClusterPlus: a class discovery tool with confidence assessments and item tracking. Bioinforma. Oxf. Engl..

[32] Kolde R. (2019). pheatmap: Pretty Heatmaps. https://CRAN.R-project.org/package=pheatmap.

[33] Li T., Fu J., Zeng Z., Cohen D., Li J., Chen Q., Li B., Liu X.S. (2020). TIMER2.0 for analysis of tumor-infiltrating immune cells. Nucleic Acids Res..

[34] Aran D., Hu Z., Butte A.J. (2017). xCell: digitally portraying the tissue cellular heterogeneity landscape. Genome Biol..

[35] Hänzelmann S., Castelo R., Guinney J. (2013). GSVA: gene set variation analysis for microarray and RNA-seq data. BMC Bioinf..

[36] Ritchie M.E., Phipson B., Wu D., Hu Y., Law C.W., Shi W., Smyth G.K. (2015). limma powers differential expression analyses for RNA-sequencing and microarray studies. Nucleic Acids Res..

[37] Love M.I., Huber W., Anders S. (2014). Moderated estimation of fold change and dispersion for RNA-seq data with DESeq2. Genome Biol..

[38] Hao Y., Hao S., Andersen-Nissen E., Mauck W.M., Zheng S., Butler A., Lee M.J., Wilk A.J., Darby C., Zager M. (2021). Integrated analysis of multimodal single-cell data. Cell.

[39] Aibar S., González-Blas C.B., Moerman T., Huynh-Thu V.A., Imrichova H., Hulselmans G., Rambow F., Marine J.-C., Geurts P., Aerts J. (2017). SCENIC: single-cell regulatory network inference and clustering. Nat. Methods.

[40] Blanche P., Dartigues J.-F., Jacqmin-Gadda H. (2013). Estimating and comparing time-dependent areas under receiver operating characteristic curves for censored event times with competing risks. Stat. Med..

[41] Dobin A., Davis C.A., Schlesinger F., Drenkow J., Zaleski C., Jha S., Batut P., Chaisson M., Gingeras T.R. (2013). STAR: ultrafast universal RNA-seq aligner. Bioinforma. Oxf. Engl..

[42] Anders S., Pyl P.T., Huber W. (2015). HTSeq--a Python framework to work with high-throughput sequencing data. Bioinforma. Oxf. Engl..

[43] Goeman J.J. (2010). L1 penalized estimation in the Cox proportional hazards model. Biom. J..

[44] Mounir M., Lucchetta M., Silva T.C., Olsen C., Bontempi G., Chen X., Noushmehr H., Colaprico A., Papaleo E. (2019). New functionalities in the TCGAbiolinks package for the study and integration of cancer data from GDC and GTEx. PLoS Comput. Biol..

[45] Colaprico A., Silva T.C., Olsen C., Garofano L., Cava C., Garolini D., Sabedot T.S., Malta T.M., Pagnotta S.M., Castiglioni I. (2016). TCGAbiolinks: an R/Bioconductor package for integrative analysis of TCGA data. Nucleic Acids Res..

[46] Silva T.C., Colaprico A., Olsen C., D’Angelo F., Bontempi G., Ceccarelli M., Noushmehr H. (2016). TCGA Workflow: Analyze cancer genomics and epigenomics data using Bioconductor packages. F1000Res..

[47] Subramanian A., Tamayo P., Mootha V.K., Mukherjee S., Ebert B.L., Gillette M.A., Paulovich A., Pomeroy S.L., Golub T.R., Lander E.S., Mesirov J.P. (2005). Gene set enrichment analysis: a knowledge-based approach for interpreting genome-wide expression profiles. Proc. Natl. Acad. Sci. USA.

[48] Kanehisa M., Goto S. (2000). KEGG: kyoto encyclopedia of genes and genomes. Nucleic Acids Res..

[49] Wolock S.L., Lopez R., Klein A.M. (2019). Scrublet: Computational Identification of Cell Doublets in Single-Cell Transcriptomic Data. Cell Syst..

[50] Robins J.M., Hernán M.A., Brumback B. (2000). Marginal structural models and causal inference in epidemiology. Epidemiol. Camb. Mass.

