# Supplementary material for: Genomic biomarkers of immunotherapy plus chemotherapy in patients with advanced NSCLC: Insights from the phase 3 ORIENT-11 study
Source: iScience. 2026 Jan 19;29(2):114730. doi: 10.1016/j.isci.2026.114730 (PMC12876322; doi:10.1016/j.isci.2026.114730)
Supplement: Document S1. Figures S1–S17 and Tables S1, S2, and S4–S8 [file mmc1.pdf]

iScience, Volume *29*

Supplemental information

Genomic biomarkers of immunotherapy plus chemotherapy in patients with advanced NSCLC: Insights from the phase 3 ORIENT-11 study

Jun Liao, Jie Huang, Xueyuan Chen, Shaodong Hong, Gang Chen, Yaxiong Zhang, Ting Zhou, Weitao Zhuang, Lanlan Pang, Yunpeng Yang, Li Zhang, and Wenfeng Fang

Supplemental information

This file includes Figures S1-S17, Table S1-S2, and Table S4-S8.

Figure S1. Study Design of ORIENT-11(related to Figure 1).

Figure S2: Identification of Hub Gene Modules Related to ICI-Chemo Efficacy (related to Figure 1).

Figure S3. Consensus Clustering Based on 45 Efficacy-Related Hub Genes (related to Figure 1).

Figure S4. Generation of Lasso Regression Genes (related to Figure 2).

Figure S5. Impact of ICPscore Genes on Clinical Outcomes in ORIENT-11 Study (related to Figure 2).

Figure S6. Flowchart of ICPscore Development Based on Gene Expression and Efficacy Data from the ORIENT-11 Study (related to Figure 2).

Figure S7. Best Overall Response of ICPscore Subgroup According to Treatment Arms in the ORIENT-11 Study (related to Figure 2).

Figure S8. Survival Analyses by ICPscore in the ORIENT-11 Study (related to Figure 2).

Figure S9. Survival Outcome by ICPscore in the OAK Study (related to Figure 3).

Figure S10. Immune subtype distribution and survival analysis in IMvigor210 cohort stratified by ICPscore (related to Figure 3).

Figure S11. Predictive Performance of the ICPscore for Immunotherapy and Chemotherapy Benefit (related to Figure 3).

Figure S12. Relationship Between PD-L1 Expression and ICPscore (related to Figure 4).

Figure S13. Impact of Predictive Biomarkers on Clinical Outcomes in ORIENT-11 Study (related to Figure 4).

Figure S14. Immunologic Features of ICPscore Subgroups in the OAK Cohort

(related to Figure 5).

Figure S15. Immunologic Features of ICPscore Subgroups in the TCGA Cohort (related to Figure 5).

Figure S16. Visualization of GOBP Term Similarity and Hierarchical Clustering (related to Figure 6).

Figure S17: Identification of cell types and differential expression analysis of myeloid cells (related to Figure 7).

Table S1. Baseline characteristics of biomarker evaluable population and biomarker non-evaluable population in ORIENT-11 study, related to STAR Methods.

Table S2. Baseline characteristics and efficacy profiles of biomarker evaluable population in ORIENT-11 study, related to STAR Methods.

Table S4. Annotation of 9 genes in ICPscore and the expression distribution by treatment arms, related to Figure 2.

Table S5. Multivariate Cox proportional hazard interaction test between treatment arm and ICPscore, related to Figure 2.

Table S6. Baseline characteristics of ORIENT-11 trial between treatment arms within ICPscore subgroup, related to Figure 2.

Table S7. Baseline characteristics of OAK trial between treatment arms within ICPscore subgroup, related to Figure 3.

Table S8. Gene list of gene signatures related to Figure 4.

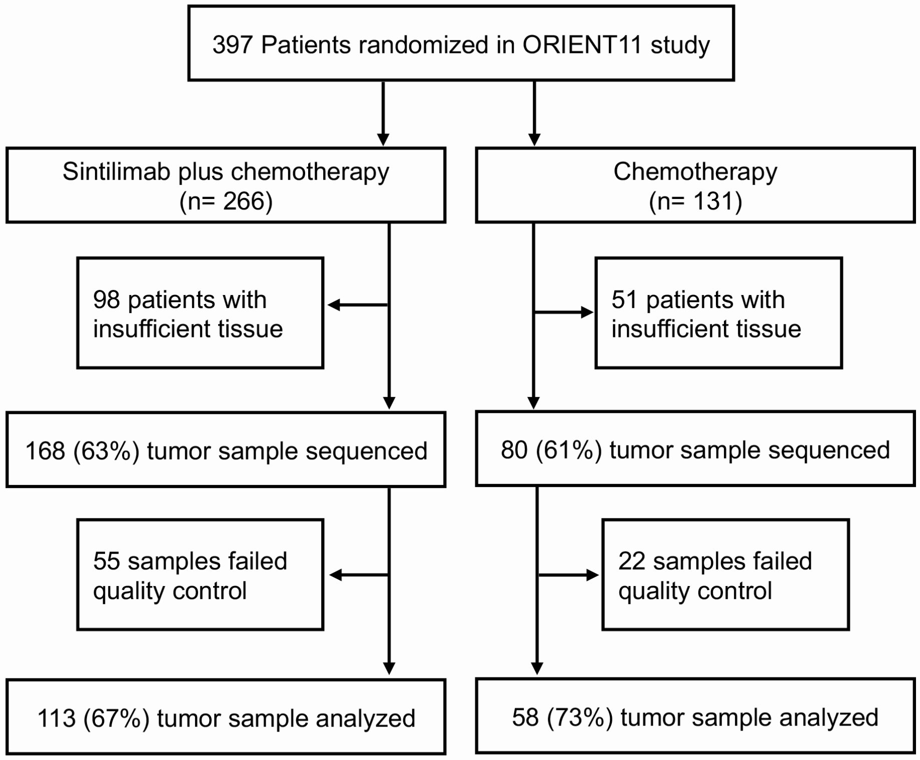

Figure S1. Study Design of ORIENT-11(related to Figure 1).

Treatment-naïve patients with advanced non-squamous NSCLC (N=397) were randomized 2:1 to receive sintilimab (n=266) or placebo (n=131), both combined with pemetrexed and platinum-based chemotherapy. Tumor samples were collected from 248 patients (62.5% of the ITT population), with 168 from the sintilimab-combination group and 80 from the chemotherapy-alone group. After quality control, 171 samples (113 sintilimab-combination, 58 chemotherapy-alone) were retained. Excluding two patients with unavailable PD-L1 TPS data, the final biomarker-evaluable population (BEP) included 169 samples (113 sintilimab-combination, 56 chemotherapy-alone), representing 42.6% of the ITT population.

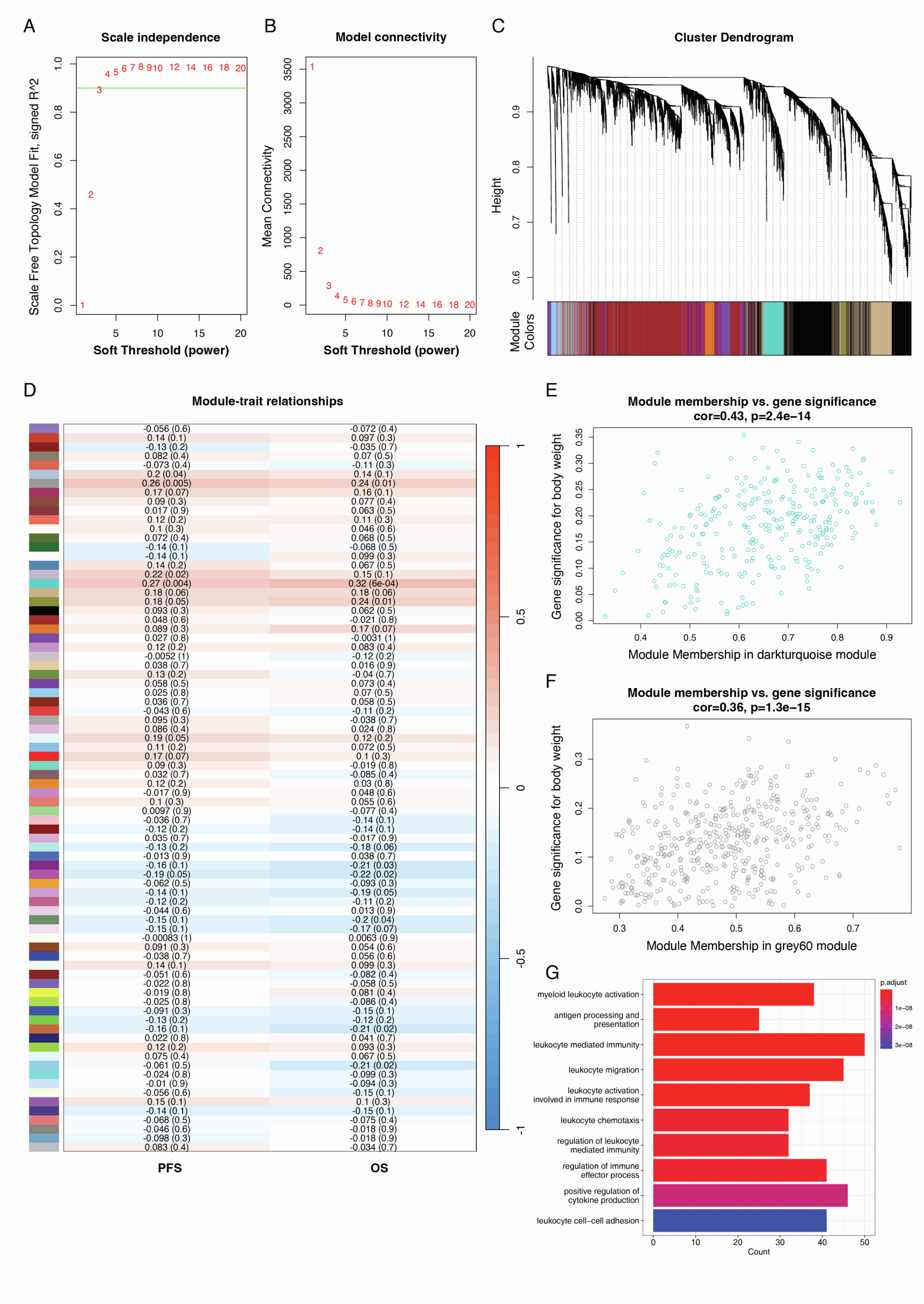

Figure S2: Identification of Hub Gene Modules Related to ICI-Chemo Efficacy (related to Figure 1).

(A) Scale-free fit index for soft threshold powers (1–20); β=3 was selected to achieve a scale-free network. (B) Average connectivity for soft threshold powers (1–20). (C) Gene clustering into 80 modules. Branches represent genes, and

genes within the same module are color-coded. (D) Module-trait relationships derived from WGCNA. (E-F) Scatter plots of genes in the darkturquoise (E) and grey60 (F) modules, with each dot representing a gene. (G) GOBP pathways enriched in the darkturquoise and grey60 modules ($p < 0.05$).

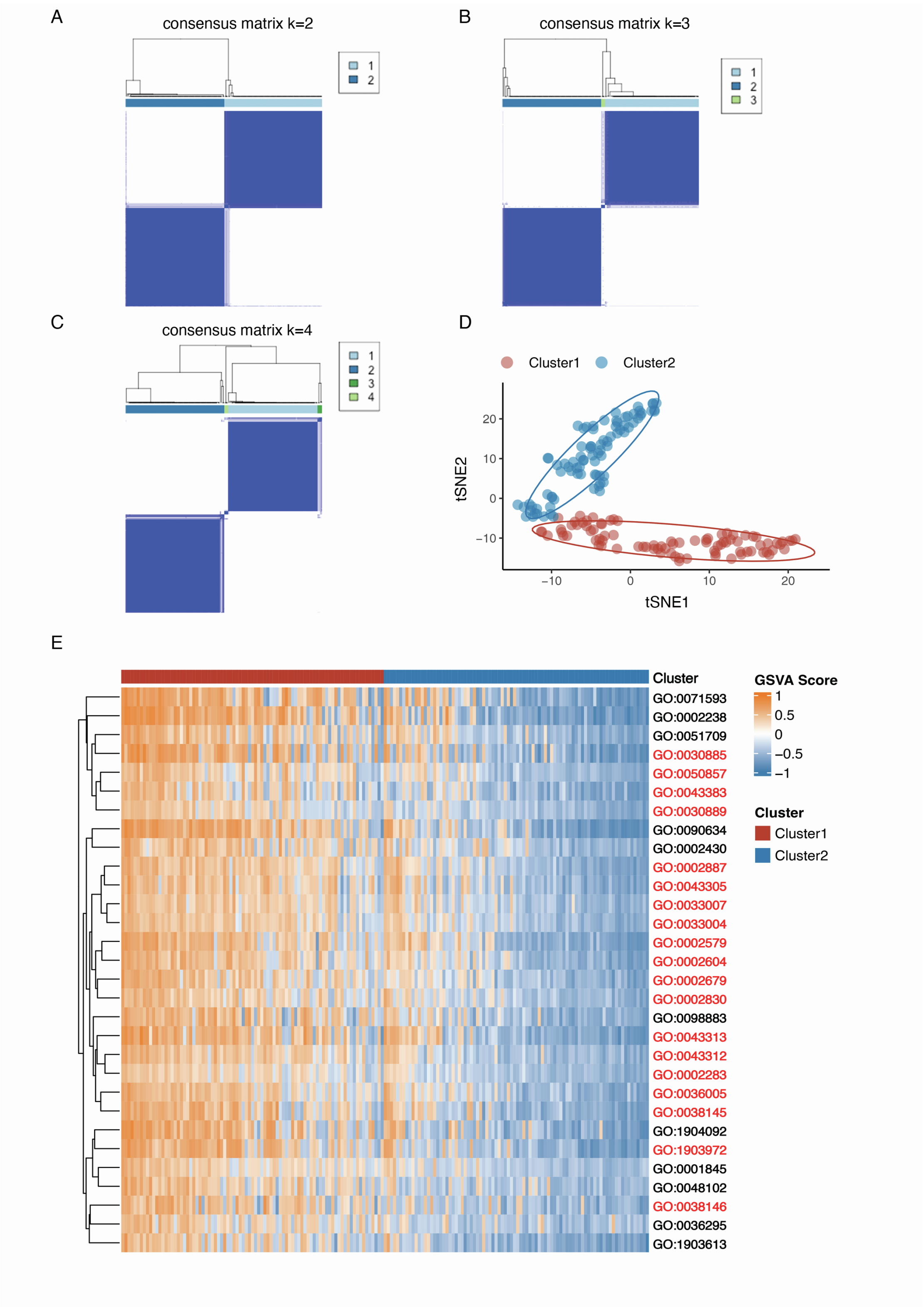

Figure S3. Consensus Clustering Based on 45 Efficacy-Related Hub Genes (related to Figure 1).

(A-C) Consensus matrices for k=2–4 in the ORIENT-11 cohort (N=171). (D) t-SNE analysis showing distinct transcriptomic profiles between the two clusters. (E) Unsupervised clustering revealed enrichment of immune activity-related biological functions in cluster 1 (highlighted in red).

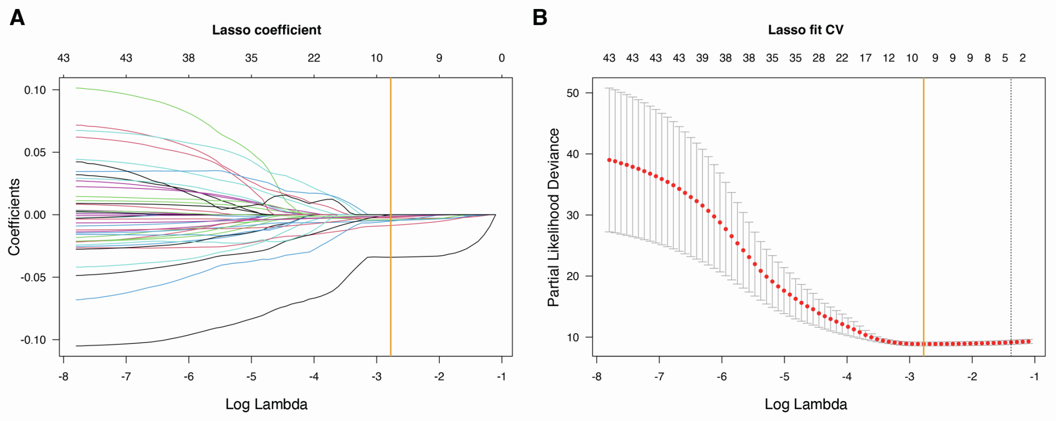

Figure S4. Generation of Lasso Regression Genes (related to Figure 2). (A) Coefficient value of the 45 hub genes. (B) Partial likelihood deviance of the 45 hub genes.

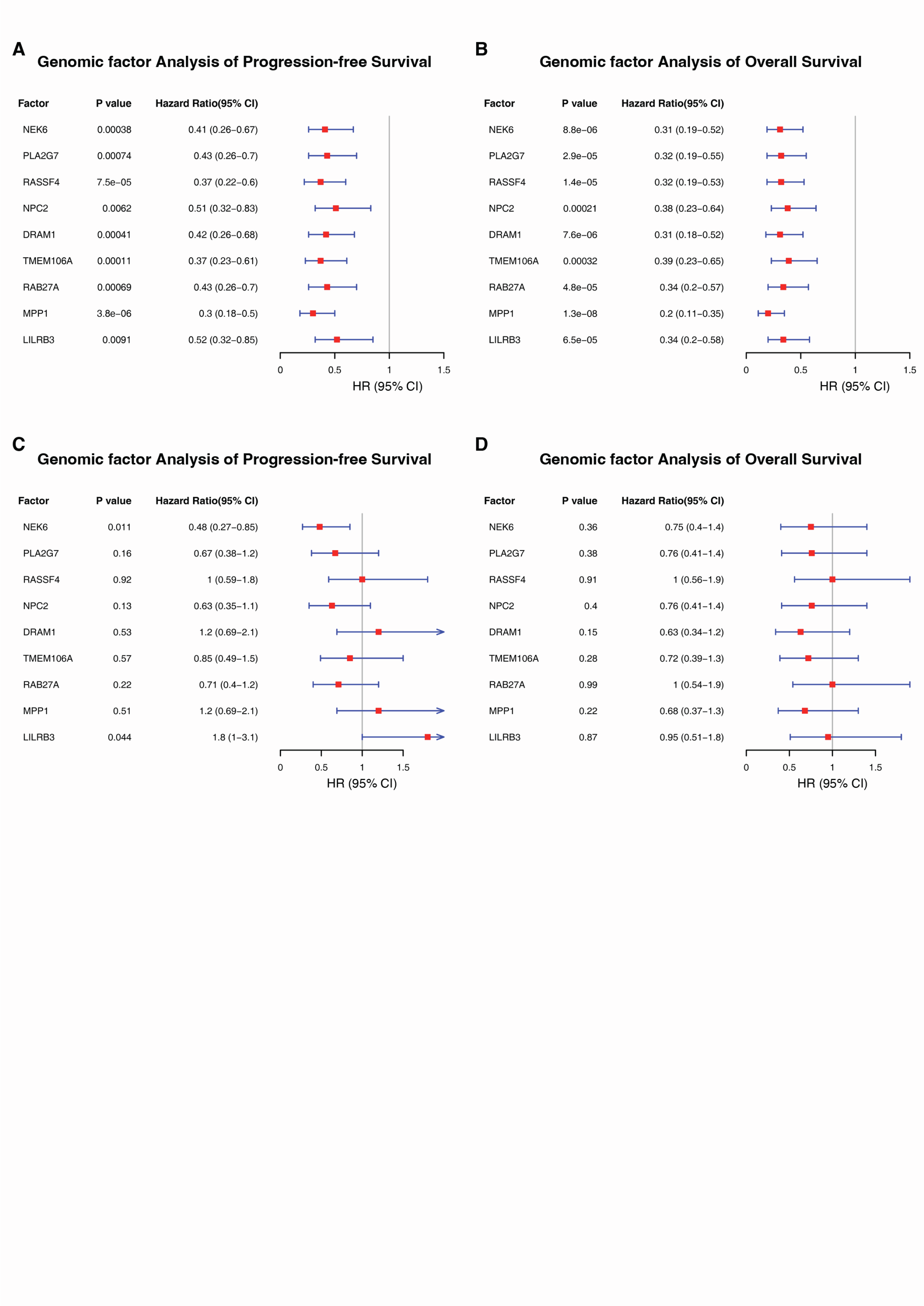

Figure S5. Impact of ICPscore Genes on Clinical Outcomes in ORIENT-11 Study (related to Figure 2).

(A-B) PFS and OS stratified by gene expression levels (high vs. low) in the ICI-Chemo cohort. (C-D) PFS and OS stratified by gene expression levels (high vs. low) in the chemotherapy cohort.

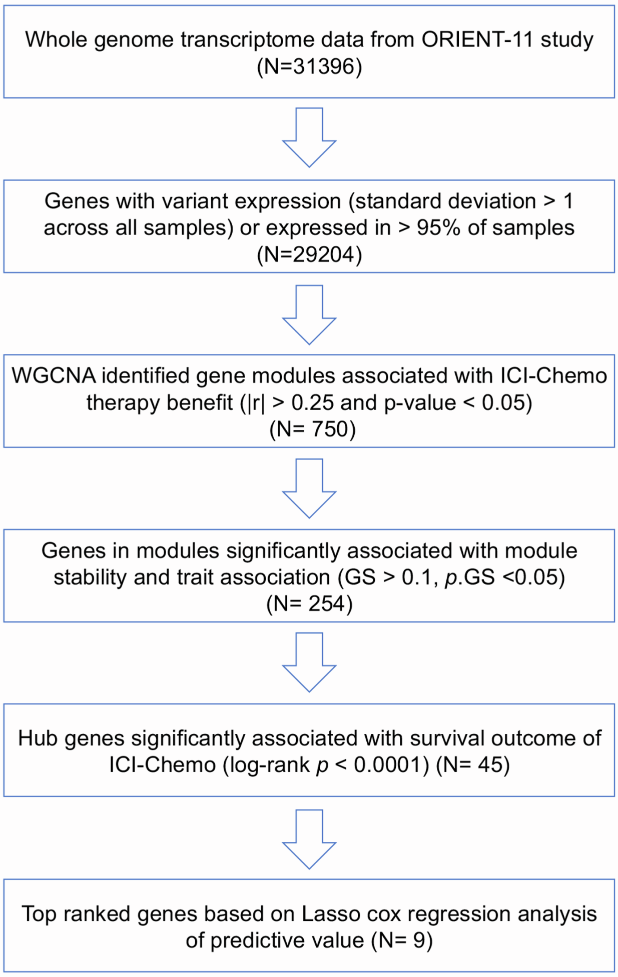

Figure S6. Flowchart of ICPscore Development Based on Gene Expression and Efficacy Data from the ORIENT-11 Study (related to Figure 2).

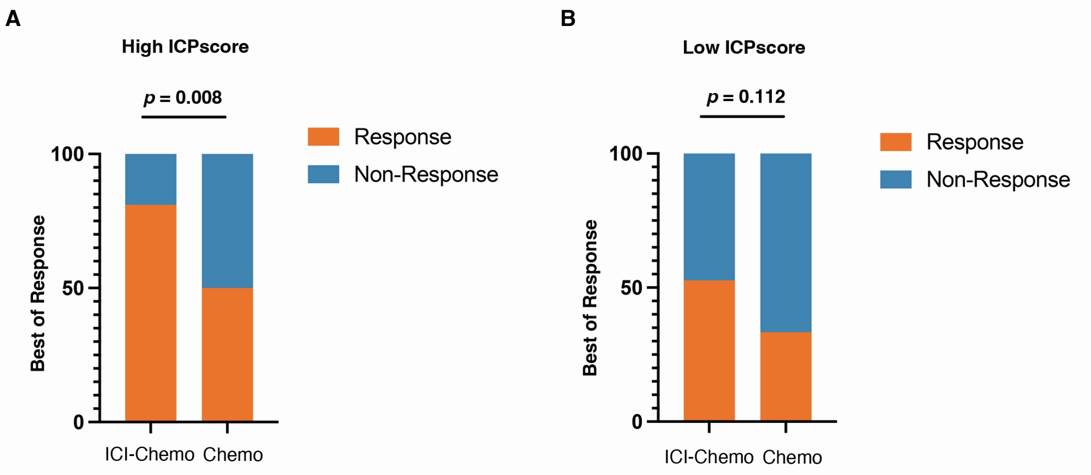

Figure S7. Best Overall Response of ICPscore Subgroup According to Treatment Arms in the ORIENT-11 Study (related to Figure 2).

(A) Best overall response in high ICPscore patients by treatment arm. (B) Best overall response in low ICPscore patients by treatment arm. Orange bars: partial or complete response; blue bars: stable or progressive diseases.

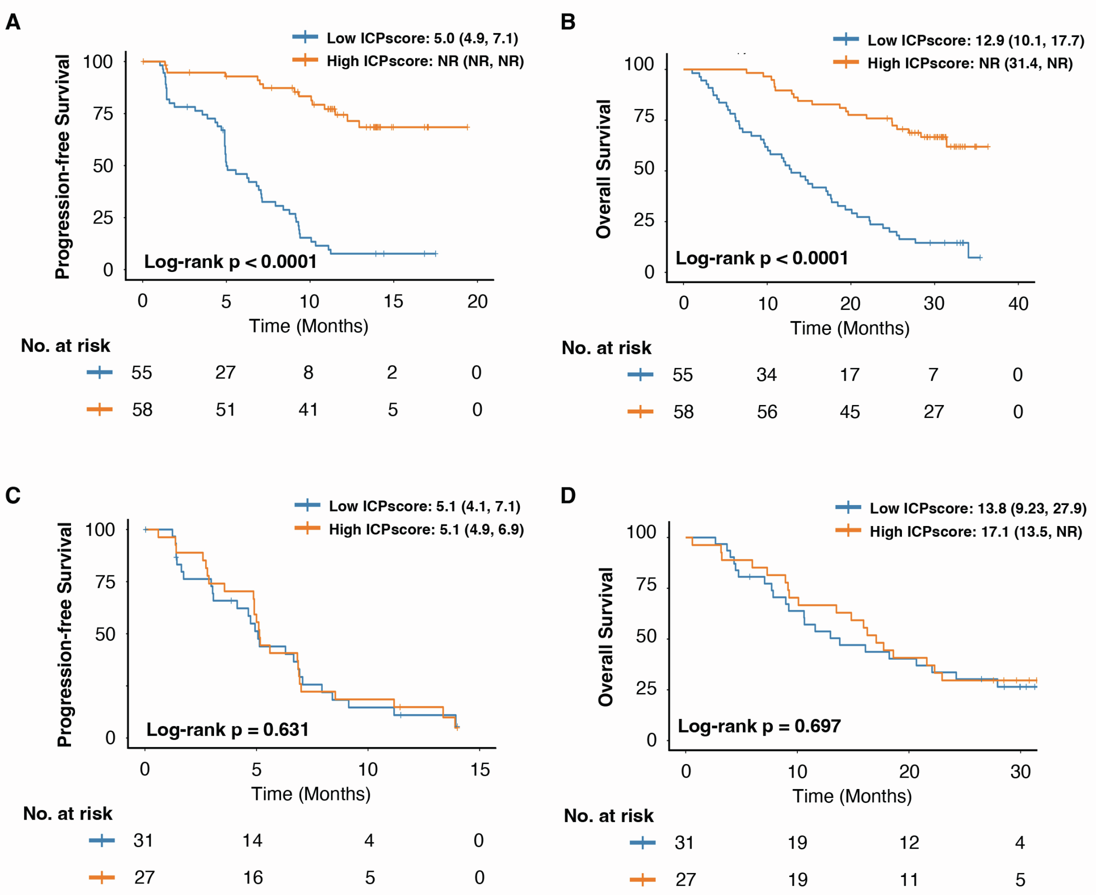

Figure S8. Survival Analyses by ICPscore in the ORIENT-11 Study (related to Figure 2).

(A-B) PFS and OS in the ICI-Chemo group stratified by ICPscore. (C-D) PFS and OS in the chemotherapy group stratified by ICPscore.

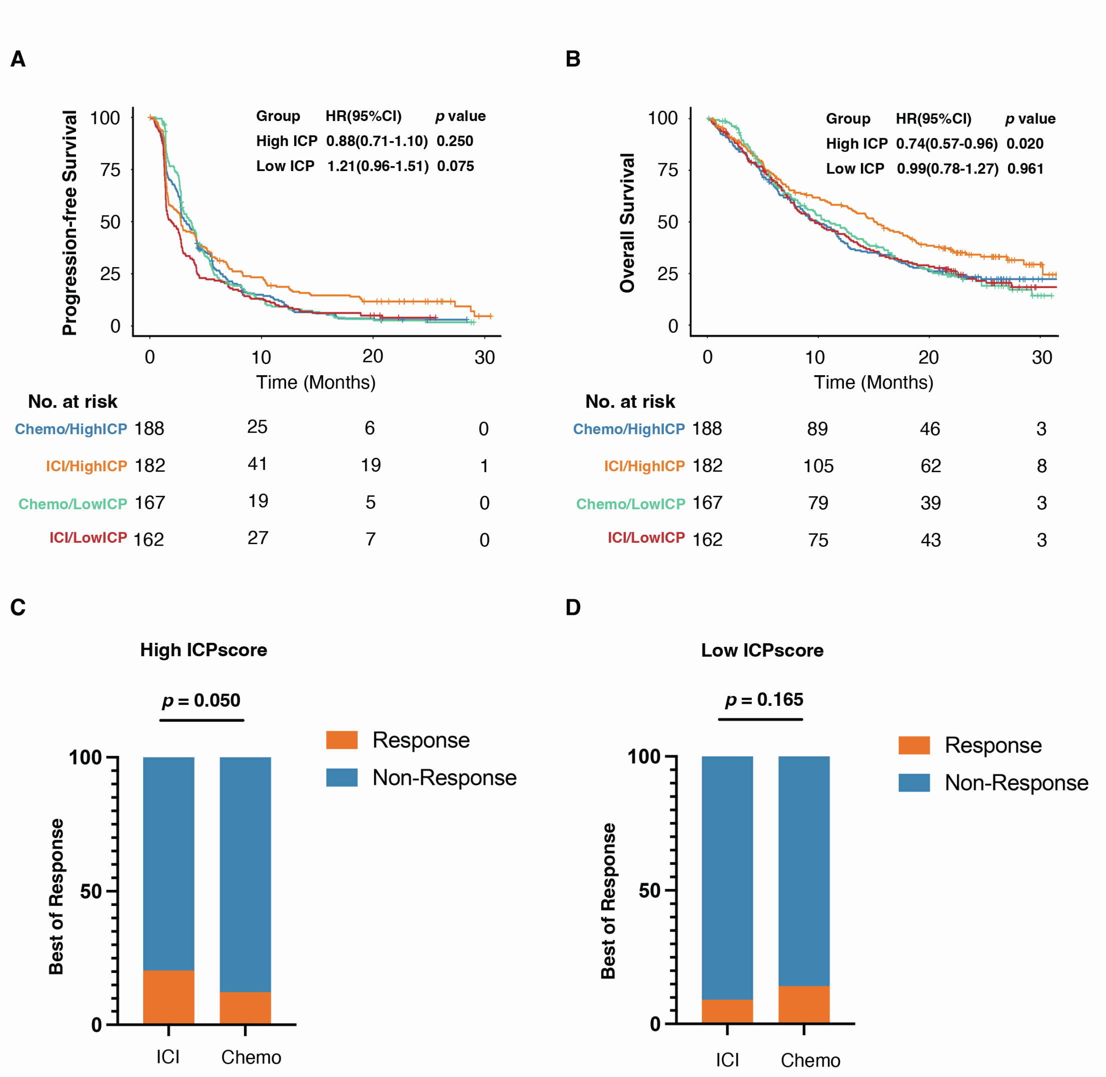

Figure S9. Survival Outcome by ICPscore in the OAK Study (related to Figure 3).

(A) PFS for ICI vs. chemotherapy stratified by ICPscore. (B) OS for ICI vs. chemotherapy stratified by ICPscore. (C-D) Best overall response in high (C) and low (D) ICPscore patients by treatment arm. Orange bars: partial or complete response; blue bars: stable or progressive diseases.

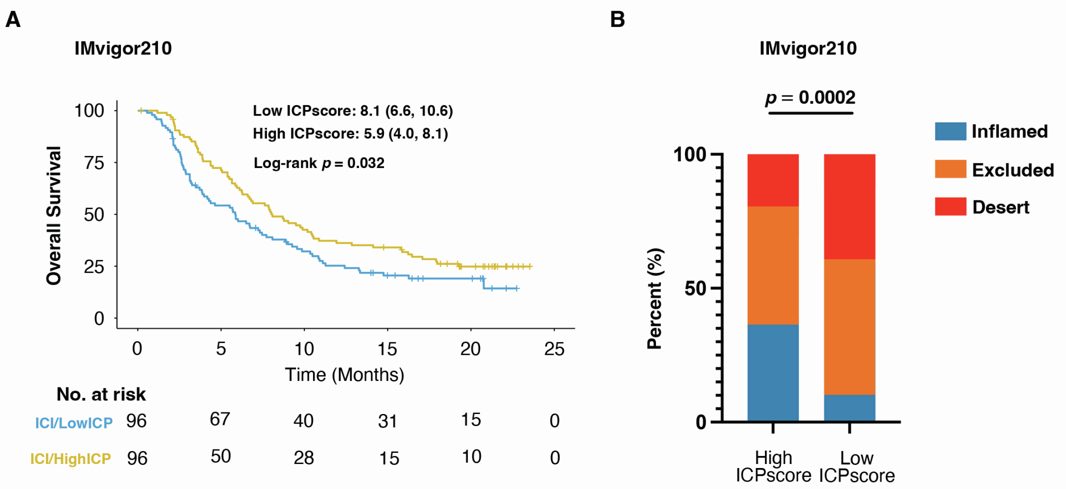

Figure S10. Immune subtype distribution and survival analysis in IMvigor210 cohort stratified by ICPscore (related to Figure 3).

(A) Kaplan-Meier curves for OS between high- and low-ICPscore groups (B) Bar plot showing the proportion of immune subtypes (inflamed, excluded, desert) in high- vs low-ICPscore groups.

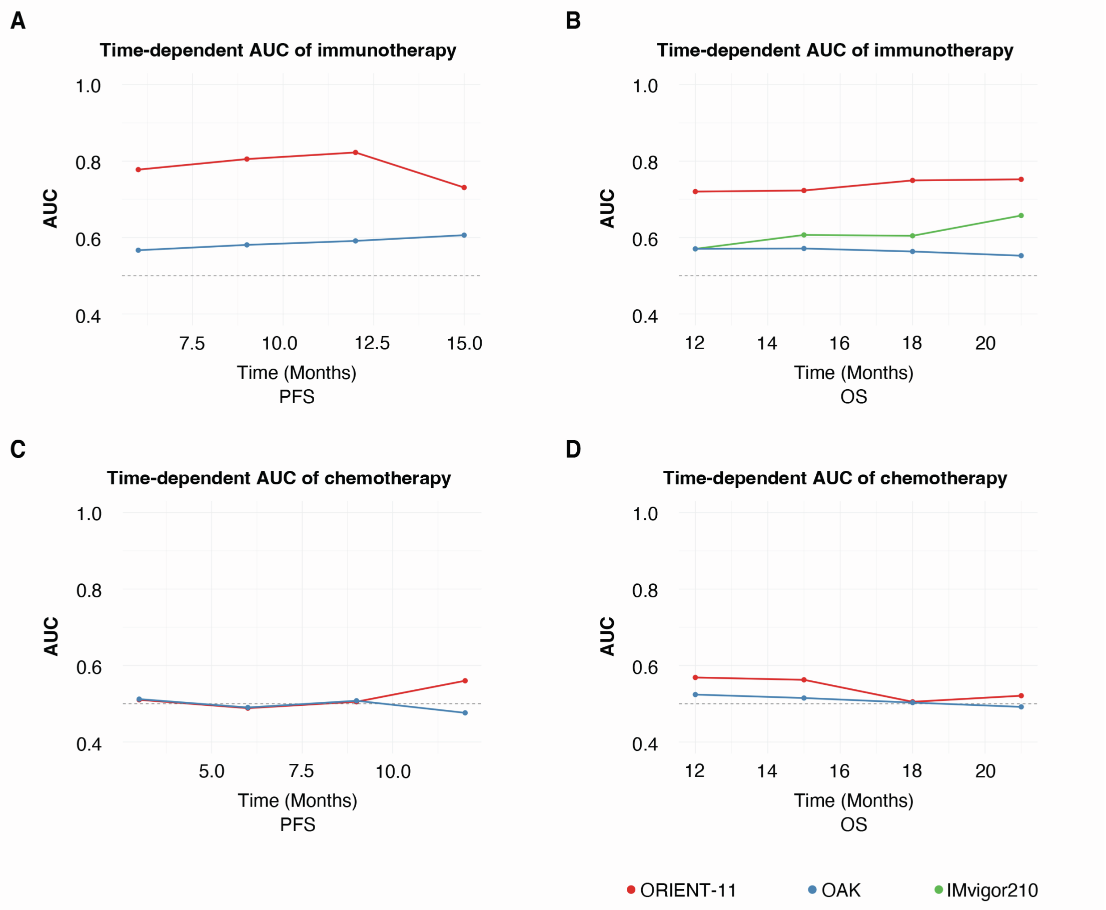

Figure S11. Predictive Performance of the ICPscore for Immunotherapy and Chemotherapy Benefit (related to Figure 3).

Time-dependent ROC curves assessing the ICPscore's predictive accuracy, as measured by the area under the curve (AUC), across different treatment modalities: (A) PFS in immunotherapy cohorts (Orient-11 [ICI-Chemo] and OAK [ICI]), (B) OS in immunotherapy cohorts (Orient-11, OAK, IMvigor210 [ICI]), (C)

PFS in chemotherapy cohorts (Orient-11 and OAK), and (D) OS in chemotherapy cohorts (Orient-11 and OAK).

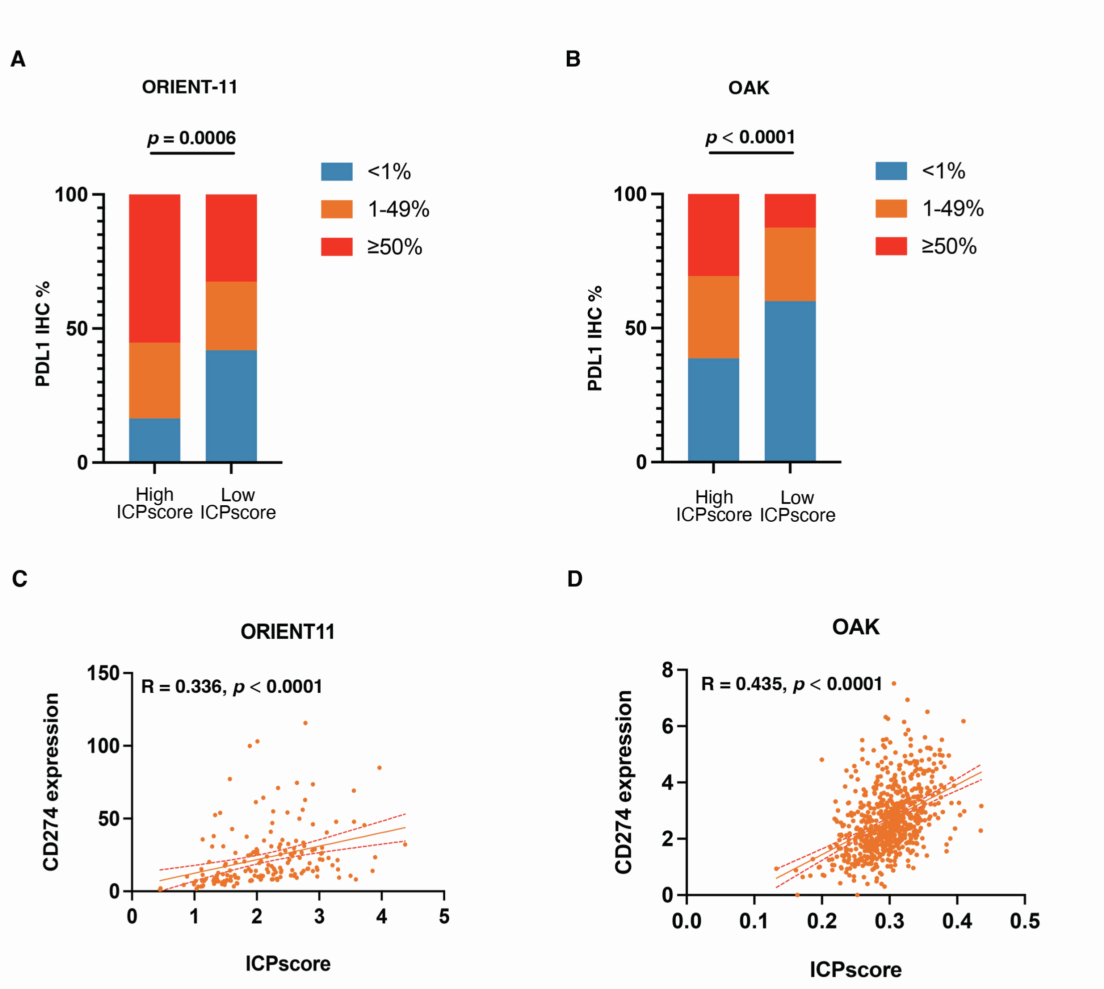

Figure S12. Relationship Between PD-L1 Expression and ICPscore (related to Figure 4).

(A-B) Distribution of PD-L1 expression (≥50%, 1-49%, <1%) in high vs. low ICPscore tumors in ORIENT-11 (A) and OAK (B) Study. (C-D) Correlation between PD-L1 mRNA expression and ICPscore in ORIENT-11 (C) and OAK (D) Study.

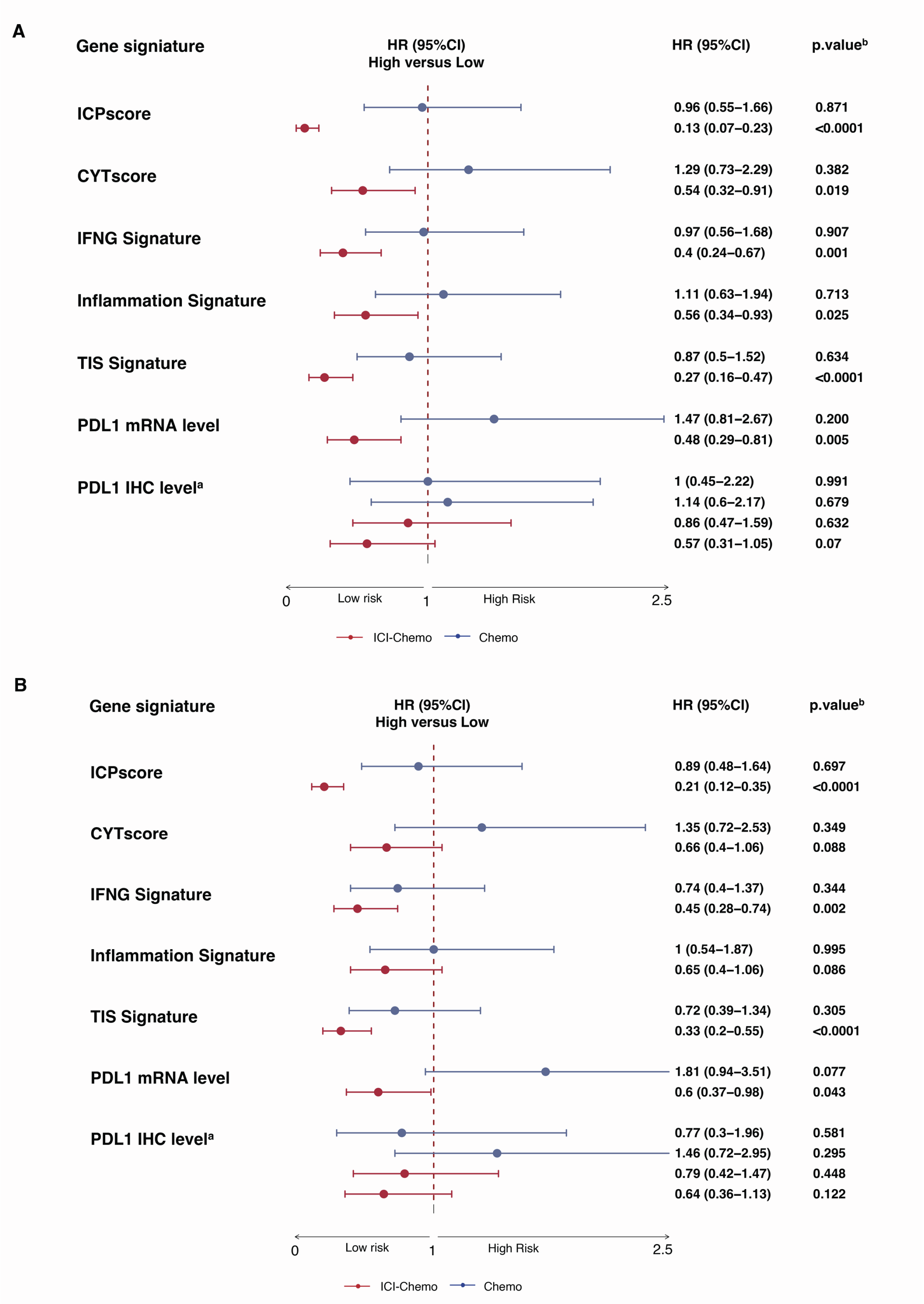

Figure S13. Impact of Predictive Biomarkers on Clinical Outcomes in ORIENT-11 Study (related to Figure 4).

(A-B) PFS (A) and OS (B) stratified by predictive biomarkers. Model with <median as the reference group was used to calculate HR and 95% CI. HR < 1 indicates better survival in the ≥median group, while HR > 1 indicates better survival in the <median group.

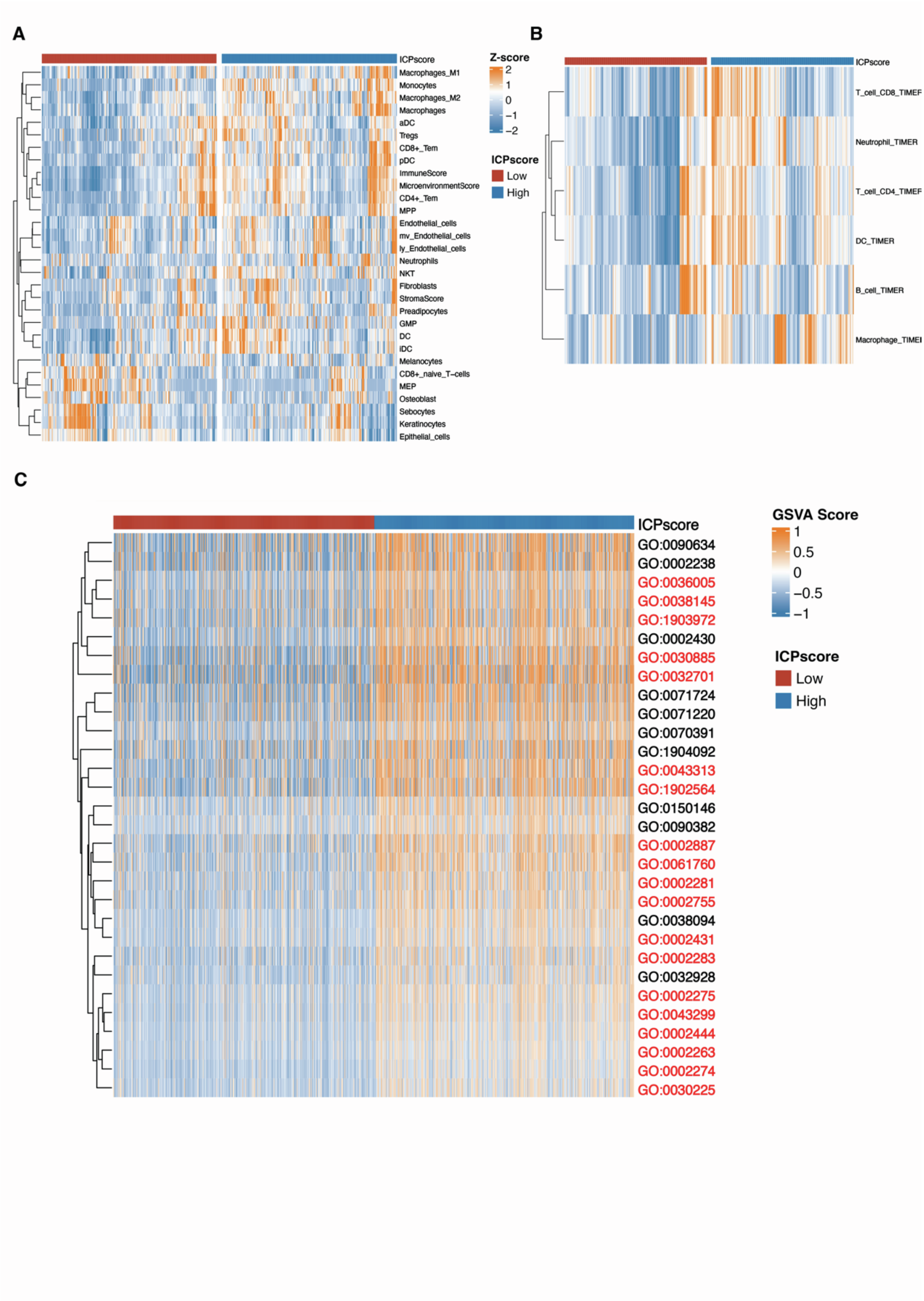

Figure S14. Immunologic Features of ICPscore Subgroups in the OAK Cohort (related to Figure 5).

(A-B) Enhanced immune cell infiltration in high ICPscore subgroups (Xcell: A; Timer: B). (C) Unsupervised clustering showing enrichment of immune-related biological functions in the high ICPscore group (highlighted in red).

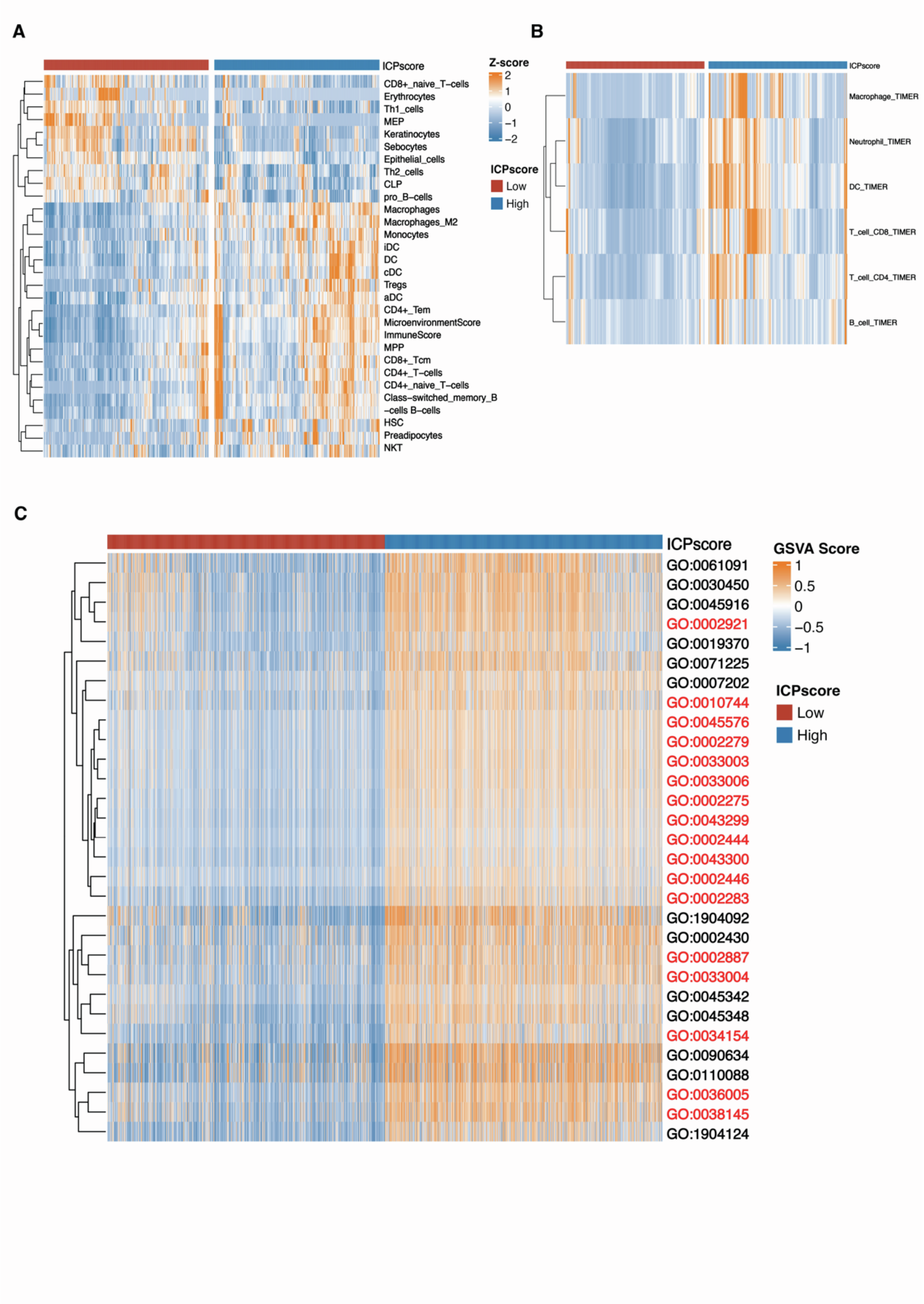

Figure S15. Immunologic Features of ICPscore Subgroups in the TCGA Cohort (related to Figure 5).

(A-B) Enhanced immune cell infiltration in high ICPscore subgroups (Xcell: A; Timer: B). (C) Unsupervised clustering showing enrichment of immune-related biological functions in the high ICPscore group (highlighted in red).

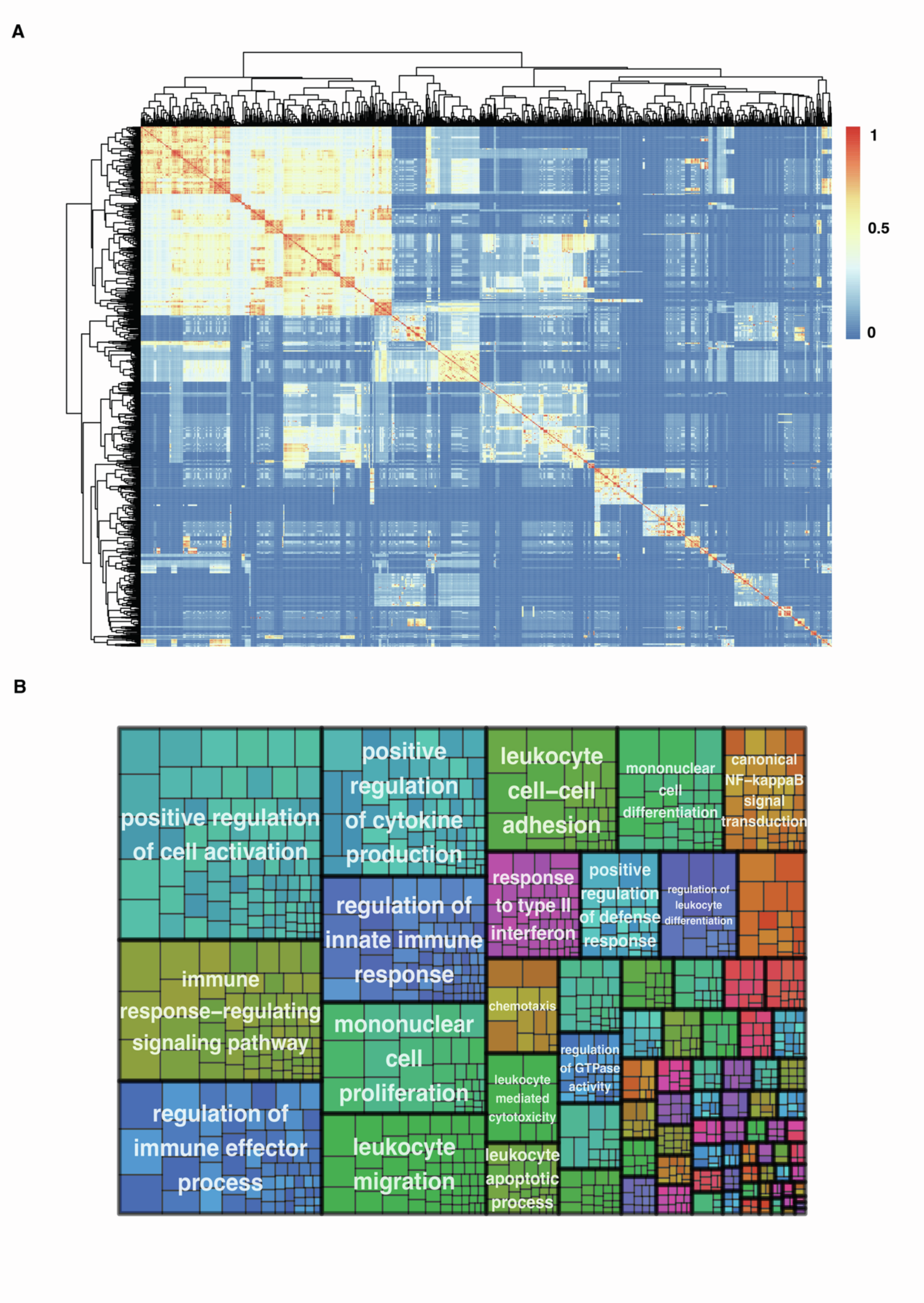

Figure S16. Visualization of GOBP Term Similarity and Hierarchical Clustering (related to Figure 6).

(A) Heatmap of GOBP term similarity matrix. Rows and columns represent GOBP terms, with color intensity indicating semantic similarity. Hierarchical clustering was applied to rows and columns. (B) Treemap of summarized GOBP terms. Terms are grouped and color-coded by parent categories, with

rectangle area proportional to enrichment scores. This visualization facilitates interpretation and comparison across GOBP term sets.

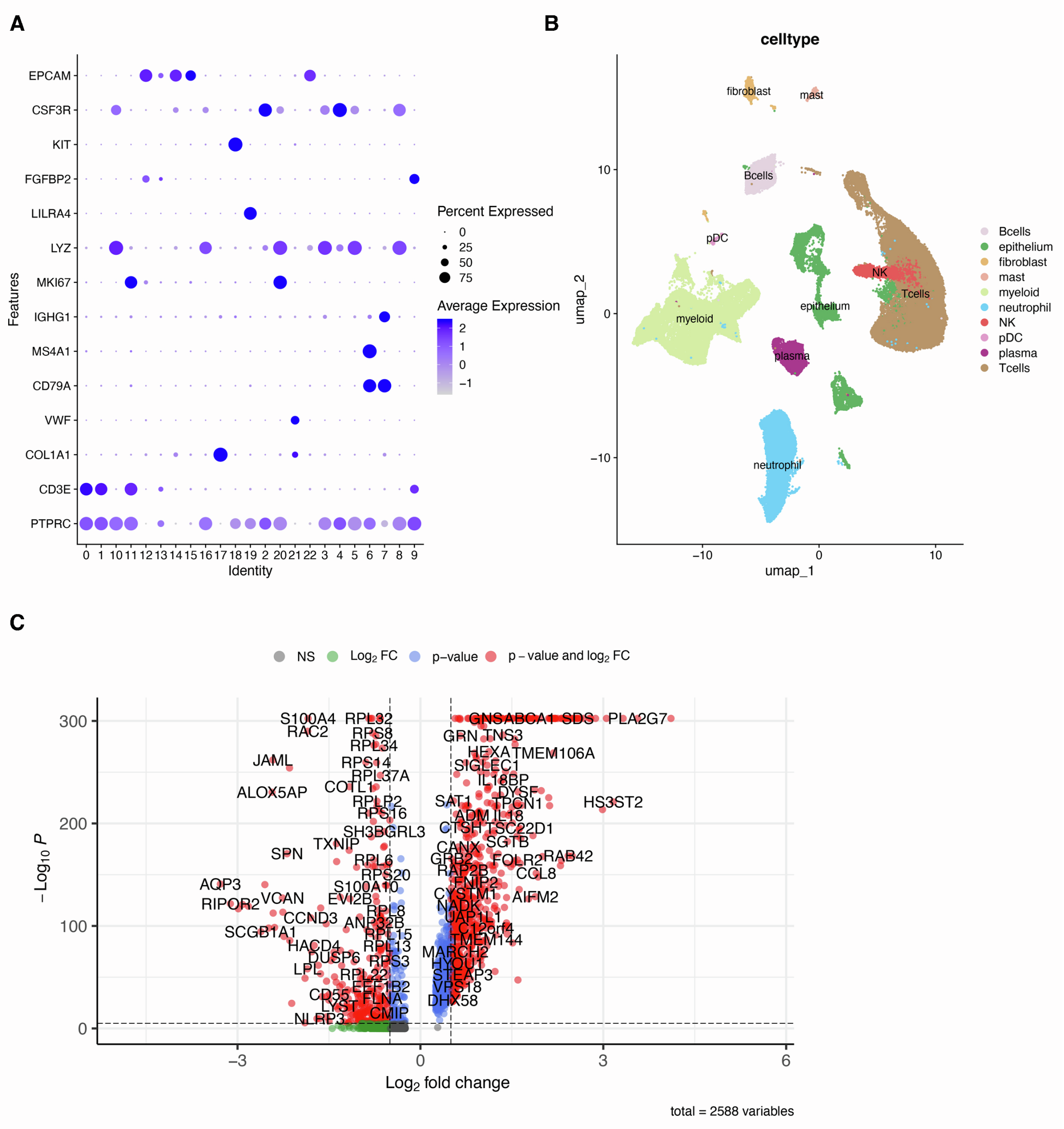

Figure S17. Identification of cell types and differential expression analysis of myeloid cells (related to Figure 7).

(A) Markers used for cell identification. (B) UMAP plots of all single cells colored by cell type. (C) Volcano plot of DEGs (|log2FC|>0.5, FDR<0.05) between high- and low-AUCell score myeloid subsets.

Table S1. Baseline characteristics of biomarker evaluable population and biomarker non-evaluable population in ORIENT-11 study, related to STAR Methods.

	BEP (n=171)	BNEP (n=226)	*P value*
**Baseline Characteristics**			
**Age, median (range), y**	61 (54-65)	61 (54-67)	0.30
**Sex, n (%)**			0.81
**Female**	39 (22.8)	55 (24.3)	
**Male**	132 (77.2)	171 (75.7)	
**BMI, mean (SD)**	23.2 (3.2)	22.7 (3.2)	0.08
**ECOG PS, n (%)**			0.35
**0**	52 (30.4)	58 (25.7)	
**1**	119 (69.6)	168 (74.3)	
**Smoking status, n (%)**			0.77
**Never**	58 (33.9)	81 (35.8)	
**Ever**	113 (66.1)	145 (64.2)	
**PD-L1 expression, n (%)**			0.47
**<1%**	50 (29.2)	79 (35.0)	
**≥1 to <50%**	46 (26.9)	54 (23.9)	
**≥50%**	75 (43.9)	93 (41.1)	
**Treatment, n (%)**			0.82
**Chemotherapy**	58 (33.9)	73 (32.3)	
**Sintilimab plus chemotherapy**	113 (66.1)	153 (67.7)	

Abbreviations: BEP, Biomarker evaluable population; BNEP, Biomarker non-evaluable population; BMI, body mass index; ECOG PS, Eastern Cooperative Oncology Group Performance Score; PD-L1, programmed death-ligand 1.

Table S2. Baseline characteristics and efficacy profiles of biomarker evaluable population in ORIENT-11 study, related to STAR Methods.

	Sintilimab plus chemotherapy[a] (n=113)	Chemotherapy (n=58)	*P value*
**Baseline Characteristics**			
**Age, median (range), y**	61 (54-65)	60 (54-64)	0.39
**Sex, n (%)**			1
**Female**	26 (23.0)	13 (22.4)	
**Male**	87 (77.0)	43 (77.6)	
**BMI, mean (SD)**	23.1 (3.0)	23.5 (3.6)	0.36
**ECOG PS, n (%)**			0.96
**0**	35 (31.0)	17 (29.3)	
**1**	78 (69.0)	41 (70.7)	
**Smoking status, n (%)**			0.28
**Never**	42 (37.2)	16 (27.6)	
**Ever**	71 (62.8)	42 (72.4)	
**PD-L1 expression, n (%)**			0.59
**<1%**	31 (27.4)	19 (32.8)	
**≥1 to <50%**	33 (29.2)	13 (22.4)	
**≥50%**	49 (43.4)	26 (44.8)	
**Efficacy profiles**			
**ORR, % (95% CI)**	67.3 (58.2-75.2)	41.1 (29.2-54.1)	0.0016
**Median PFS, months (95% CI)**	9.4 (8.4-12.9)	5.1 (4.9-6.9)	<0.001
**Median OS, months (95% CI)**	23.8 (18.4-31.4)	16.1 (11.6-22.3)	0.053

Abbreviations: BMI, body mass index; ECOG PS, Eastern Cooperative Oncology Group Performance Score; PD-L1, programmed death-ligand 1; ORR, overall response rate; PFS, progression-free survival; OS, overall survival;

[a] Sintilimab is anti-PD-1 antibody and chemotherapy refer to platinum-based chemotherapy.

Table S4. Annotation of 9 genes in ICPscore and the expression distribution by treatment arms, related to Figure 2.

Gene	Annotation	Expression in combination arm, mean TPM (SD)	Expression in chemotherapy arm, mean TPM (SD)
NEK6	Protein kinase which plays an important role in mitotic cell cycle progression (PubMed: 11516946, 14563848). Required for chromosome segregation at metaphase-anaphase transition, robust mitotic spindle formation and cytokinesis (PubMed: 19414596). Involved in G2/M phase cell cycle arrest induced by DNA damage (PubMed: 18728393). Inhibition of activity results in apoptosis. May contribute to tumorigenesis by suppressing p53/TP53-induced cancer cell senescence (PubMed: 21099361).	163.76 (56.72)	158.72 (54.13)
PLA2G7	Lipoprotein-associated calcium-independent phospholipase A2 involved in phospholipid catabolism during inflammatory and oxidative stress response (PubMed: 10066756, 16371369, 17090529, 2040620, 7700381, 8624782).	40.43 (29.29)	37.64 (26.05)
RASSF4	Potential tumor suppressor. May act as a KRAS effector protein. May promote apoptosis and cell cycle arrest. (RASF4_HUMAN, Q9H2L5)	70.95 (42.41)	69.90 (50.52)
NPC2	Intracellular cholesterol transporter which acts in concert with NPC1 and plays an important role in the egress of cholesterol from the lysosomal compartment (PubMed: 11125141, 15937921, 17018531, 18772377, 29580834).	121.61 (107.01)	94.57 (75.86)

DRAM1	Lysosomal modulator of autophagy that plays a central role in p53/TP53-mediated apoptosis. Not involved in p73/TP73-mediated autophagy. (DRAM1_HUMAN, Q8N682)	82.13 (59.34)	77.93 (54.55)
TMEM106A	Activates macrophages and polarizes them into M1-like macrophages through the activation of the MAPK and NF-kappaB signaling pathway. Upon activation, up-regulates the expression of CD80, CD86, CD69 and MHC II on macrophages, and induces the release of pro-inflammatory cytokines such as TNF, IL1B, IL6, CCL2 and nitric oxide (By similarity). May play a role in inhibition of proliferation and migration (PubMed: 29131025, 30456879).	51.03 (18.25)	49.10 (21.56)
RAB27A	Small GTPase which cycles between active GTP-bound and inactive GDP-bound states. In its active state, binds to a variety of effector proteins to regulate homeostasis of late endocytic pathway, including endosomal positioning, maturation and secretion (PubMed:30771381). Plays a role in cytotoxic granule exocytosis in lymphocytes. Required for both granule maturation and granule docking and priming at the immunologic synapse. (RB27A_HUMAN, P51159)	58.63 (24.88)	55.82 (22.54)
MPP1	Essential regulator of neutrophil polarity. Regulates neutrophil polarization by regulating AKT1 phosphorylation through a mechanism that is independent of	13.06 (5.93)	14.55 (7.85)

	PIK3CG activity (By similarity). (EM55_HUMAN, Q00013)		
LILRB3	May act as receptor for class I MHC antigens. Becomes activated upon coligation of LILRB3 and immune receptors, such as FCGR2B and the B-cell receptor. Down-regulates antigen-induced B-cell activation by recruiting phosphatases to its immunoreceptor tyrosine-based inhibitor motifs (ITIM). (LIRB3_HUMAN, O75022)	16.05 (9.99)	15.97 (9.21)

Abbreviations: TPM, Transcripts Per Million; SD, standard deviation.

Table S5. Multivariate Cox proportional hazard interaction test between treatment arm and ICPscore, related to Figure 2.

	Progression-free survival				Overall survival			
	**z**	**Pr(>|z|)**	**exp(coef)**	**95% CI**	**z**	**Pr(>|z|)**	**exp(coef)**	**95% CI**
**ORIENT-11[a]**	-4.70	<0.0001	0.15	0.07-0.33	-3.42	0.0006	0.24	0.11-0.56
**ORIENT-11[b]**	-4.87	<0.0001	0.14	0.06-0.31	-3.40	0.0007	0.24	0.10-0.54
**OAK**	-2.21	0.027	0.70	0.52-0.96	-1.58	0.115	0.75	0.53-1.07

A positive or negative standardized Wald test z-value indicates that the high ICPscore reduced or improved the efficacy of immunotherapy versus chemotherapy, respectively.

[a] Before IPW adjustment; [b] After IPW adjustment.

Table S6. Baseline characteristics of ORIENT-11 trial between treatment arms within ICPscore subgroup, related to Figure 2.

	High ICPscore			Low ICPscore		
	ICI-Chemo [a]	Chemo	P value	ICI-Chemo [a]	Chemo	P value
**No. n (%)**	58	27		55	31	
**Age, y median (range)**	60 (54-65)	62 (56-65)	0.752	63 (55-66)	59 (54-63)	0.115
**Sex**			0.487			0.627
**Female**	14 (24.1)	4 (14.8)		12 (21.8)	9 (29.0)	
**Male**	44 (75.9)	23 (85.2)		43 (78.2)	22 (71.0)	
**BMI, mean (SD)**	23.2 (2.8)	23.1 (3.5)	0.903	23.0 (3.27)	24.0 (3.57)	0.190
**ECOG PS**			0.727			0.915
**0**	21 (36.2)	8 (29.6)		14 (25.5)	9 (29.0)	
**1**	37 (63.8)	19 (70.4)		41 (74.5)	22 (71.0)	
**Smoking status**			0.701			0.344
**Non-smoker**	19 (32.8)	7 (25.9)		23 (41.8)	9 (29.0)	
**Smoker**	39 (67.2)	20 (74.1)		32 (58.2)	22 (71.0)	
**PD-L1 expression**			1.00			0.314
**<1%**	10 (17.2)	4 (14.8)		21 (38.2)	15 (48.4)	
**1-49%**	16 (27.6)	8 (29.6)		17 (30.9)	5 (16.1)	
**≥50%**	32 (55.2)	15 (55.6)		17 (30.9)	11 (35.5)	

Abbreviations: Chemo, chemotherapy; BMI, body mass index; ECOG PS, Eastern Cooperative Oncology Group Performance Score; PD-L1, programmed death-ligand 1.

[a] Sintilimab plus platinum-based chemotherapy.

Table S7. Baseline characteristics of OAK trial between treatment arms within ICPscore subgroup, related to Figure 3.

	High ICPscore			Low ICPscore		
	ICI [a]	Chemo	P value	ICI [a]	Chemo	P value
**No. n (%)**	182	167		162	188	
**Sex**			0.186			0.455
**Female**	68 (37.4)	75 (44.9)		57 (35.2)	58 (30.9)	
**Male**	114 (62.6)	92 (55.1)		105 (64.8)	130 (69.1)	
**PD-L1 expression**			0.157			0.510
**<1%**	31 (17.0)	41 (24.6)		54 (33.3)	51 (27.1)	
**1-49%**	36 (19.8)	21 (12.6)		19 (11.7)	29 (15.4)	
**≥50%**	29 (15.9)	28 (16.8)		11 (6.79)	11 (5.85)	
**Unknown**	86 (47.3)	77 (46.1)		78 (48.1)	97 (51.6)	
**Tissue TMB**			0.857			0.747
**<16**	77 (42.3)	66 (39.5)		80 (49.4)	100 (53.2)	
**≥16**	28 (15.4)	28 (16.8)		23 (14.2)	23 (12.2)	
**Unknown**	77 (42.3)	73 (43.7)		59 (36.4)	65 (34.6)	
**Histology**			0.845			0.590
**Non-squamous**	155 (85.2)	140 (83.8)		102 (63.0)	112 (59.6)	
**Squamous**	27 (14.8)	27 (16.2)		60 (37.0)	76 (40.4)	

Abbreviations: ICI, immune checkpoint inhibitor; Chemo, chemotherapy; PD-L1, programmed death-ligand 1; TMB, tumor mutation burden. For PD-L1 expression, tissue TMB, the statistical tests were conducted after removing patients with unevaluable information.

Table S8. Gene list of gene signatures related to Figure 4.

Signature	Gene list
Nine-gene ICPscore	NEK6, PLA2G7, RASSF4, NPC2, DRAM1, TMEM106A, RAB27A, MPP1, LILRB3
Cytolytic Activity (CYT) score	GZMA, PRF1
IFN-γ signaling signature	TIGIT, CD27, CD8A, PDCD1LG2, LAG3, CD274, CXCR6, CMKLR1, NKG7, CCL5, PSMB10, IDO1, CXCL9, HLA-DQA1, CD276, STAT1, HLA- DRB1, HLA-E.
4-gene Inflammation Gene Signature	CD274, CD8A, LAG3, STAT1
18-gene T cell–inflamed signature (TIS)	TIGIT, CD27, CD8A, PDCD1LG2, LAG3, CD274, CXCR6, CMKLR1, NKG7, CCL5, PSMB10, IDO1, CXCL9, HLA-DQA1, CD276, STAT1, HLA- DRB1, HLA-E.

Abbreviations: ICP, Immune-chemotherapy prediction; IFN, interferon;

Note: The cutoff was the median value of each signature.
